# Bacterial extracellular vesicles in inflammatory diseases: emerging roles and mechanisms

**DOI:** 10.1093/rb/rbag129

**Published:** 2026-06-16

**Authors:** Chun Yang, Muran Bai, Yucheng Wan, Yingying Qian, Zhongshi Li, Qing Zheng, Liqun Lv, Yan Zhang, Zhijin Fan, Hongju Yang, Yuhui Liao

**Affiliations:** College of Basic Medicine, Beihua University, Jilin 132033, China; College of Basic Medicine, Beihua University, Jilin 132033, China; Geriatric Medical Center, Division of Geriatric Gastroenterology, The First Affiliated Hospital of Kunming Medical University, Kunming 650032, China; Hospital of Stomatology, Zunyi Medical University, Zunyi 563000, China; College of Basic Medicine, Beihua University, Jilin 132033, China; Institute for Engineering Medicine, Kunming Medical University, Kunming 650500, China; College of Basic Medicine, Beihua University, Jilin 132033, China; College of Basic Medicine, Beihua University, Jilin 132033, China; School of Public Health, Beihua University, Jilin 132033, China; Institute for Engineering Medicine, Kunming Medical University, Kunming 650500, China; Geriatric Medical Center, Division of Geriatric Gastroenterology, The First Affiliated Hospital of Kunming Medical University, Kunming 650032, China; Institute for Engineering Medicine, Kunming Medical University, Kunming 650500, China

**Keywords:** extracellular vesicles, bacterial extracellular vesicles, outer membrane vesicles, inflammatory diseases, precision medicine

## Abstract

Bacterial extracellular vesicles (BEVs), nanoscale membrane-bound particles secreted by bacteria, have emerged as pivotal mediators of host–microbe communication, particularly in the context of inflammatory diseases. By delivering diverse bioactive cargos including proteins, lipids, nucleic acids and metabolites, BEVs orchestrate complex immunological responses, playing dual roles in both the exacerbation and resolution of inflammation. This review presents a comprehensive overview of the biogenesis, classification and molecular composition of BEVs, with emphasis on their immunomodulatory functions. Mechanistically, BEVs derived from pathogenic bacteria can trigger robust pro-inflammatory signaling cascades, disrupt epithelial barriers and promote tissue destruction, contributing to disease progression in conditions such as periodontitis, rheumatoid arthritis and pulmonary disorders. Conversely, BEVs from commensal and probiotic strains exhibit anti-inflammatory and tissue-reparative effects, mediated through macrophage polarization, cytokine modulation and gut microbiota homeostasis. Recent advances highlight the therapeutic potential of engineered BEVs as bio-nanocarriers for targeted drug delivery, vaccine platforms and immunotherapies. We further explore the translational challenges of BEV-based strategies, including scalability, targeting specificity and biocompatibility. By elucidating the multifaceted roles of BEVs in inflammatory pathophysiology, this review offers new insights into their application as next-generation nanotherapeutics for precision medicine.

## Graphical abstract

**Graphical Abstract:**
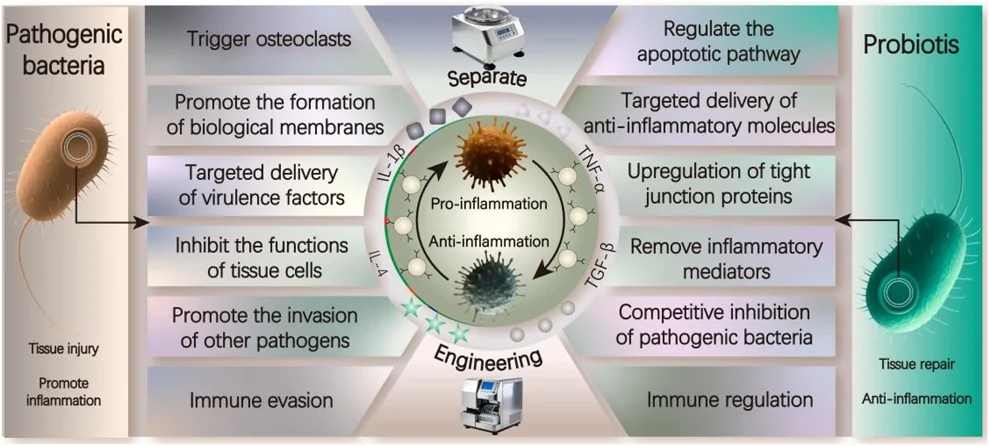

## Introduction

Extracellular vesicles (EVs) have gained considerable attention for their roles in various biological processes, particularly their mechanisms in inflammatory diseases [1–3]. Inflammation represents a defensive reaction of the organism against noxious stimuli. Such stimuli encompass pathogens, damaged cells or irritants. Inflammation assumes a vital physiological function in the maintenance of internal homeostasis. Additionally, it triggers tissue repair [4]. However, when inflammation becomes chronic or dysregulated, it can lead to tissue damage and a series of pathological changes. Bacterial extracellular vesicles (BEVs) participate in this process by delivering pathogen-associated molecular patterns (PAMPs), which bind to pattern recognition receptors (PRRs) on various cell types, including macrophages, dendritic cells (DCs), epithelial cells and even endothelial cells [5]. Receptor activation promotes the secretion of pro-inflammatory cytokines and chemokines. This incites local inflammation. Unchecked, it may become systemic [6–8]. Recent advances have revealed that BEVs are intimately involved in the pathogenesis of many inflammatory diseases. These range from chronic respiratory ailments to gastrointestinal disorders. They also include systemic conditions such as sepsis and neuroinflammatory disorders [9–13].

BEVs are nanoscale membrane-bound vesicles released by bacteria, with diameters ranging from 20 to 400 nm [14]. BEVs carry proteins, lipids, nucleic acids and metabolites. This cargo transfers to recipient cells without direct bacterial contact [5]. The cargo of BEVs exhibits remarkable diversity, which reflects the physiological state of the bacterial producer. These cargo molecules encompass classical virulence determinants, such as toxins and adhesins, as well as molecules that regulate host immune function, including nucleic acids and small regulatory RNAs [15, 16]. Upon interacting with host cells, these EVs initiate immune recognition through surface binding to receptors. These receptors encompass TLR2, TLR4, TLR7 and TLR8. Additionally, they activate intracellular pathways related to nucleotide-binding oligomerization domain (NOD)-like receptors [8, 17]. This activates downstream cascades, including NF-κB and MAPK pathways. Ultimately, this activation leads to the secretion of pro-inflammatory mediators. These mediators include interleukin (IL)-1β, IL-6 and tumor necrosis factor-α (TNF-α). They also include chemokines that coordinate recruitment of additional immune effector cells [10]. This intercellular communication allows BEVs to modulate host immunity. In turn, they influence inflammatory disease progression. Studies have shown that BEVs exhibit dual regulatory roles in inflammatory diseases [18]. Pathogenic BEVs often carry virulence factors that induce pro-inflammatory responses. This leads to tissue damage and disease progression. In contrast, BEVs from commensal or probiotic bacteria may exert anti-inflammatory effects. These effects help restore immune homeostasis and alleviate disease symptoms [19–23].

This review summarizes recent advances in EV research. We focus on mechanisms relevant to inflammatory diseases. We will delve into the biological significance of inflammation, the stages of the inflammatory cascade, as well as the origin, structural features and molecular composition of BEVs. This article will analyze the dual regulatory mechanisms of BEVs in inflammation. We will explore their potential therapeutic applications in inflammatory diseases. We will also assess the challenges and prospects for clinical translation. Better understanding of BEV-host interactions could lead to new anti-inflammatory strategies. Clinical outcomes may improve as a result.

## The overview of inflammatory diseases

Inflammation is a protective response of the body to infection, injury or harmful stimuli, characterized by acute and chronic forms. A deeper understanding of the molecular mechanisms and signaling pathways involved in inflammation is essential for advancing effective prevention and therapeutic strategies, ultimately improving the clinical management of inflammation-related diseases.

### The biological significance of inflammation

Inflammation is a highly complex and finely regulated biological response of the immune system to harmful stimuli such as pathogens, damaged cells or toxic compounds [7, 24, 25]. When the body encounters bacterial or viral invasions or cellular damage due to physical or chemical factors, the immune system rapidly initiates an inflammatory response. During this process, immune cells are activated and recruited to the site of injury. They release various inflammatory mediators. These mediators promote vasodilation and increase vascular permeability. This allows more immune cells and nutrients to reach the inflamed area, thereby effectively clearing pathogens, repairing damaged tissue and maintaining tissue homeostasis. Thus, inflammation plays a vital role in defending against infections and facilitating tissue repair, serving as a critical line of defense for the body [26].

However, the regulation of inflammation is not always ideal. In some pathological states, inflammation becomes dysregulated. It may fail to resolve or become overactive. The immune system then attacks host tissues. Such dysregulation can trigger severe health issues and contribute to the development of chronic diseases. For example, a chronic inflammatory environment may promote the proliferation, invasion and metastasis of tumor cells, creating favorable conditions for cancer progression. In arthritis patients, prolonged inflammation in the joints leads to cartilage and bone destruction, causing pain, swelling and dysfunction [27]. Cardiovascular diseases are closely linked to inflammation. Inflammation accelerates the formation and destabilization of atherosclerotic plaques. This increases the risk of thrombosis and subsequent events. Examples include myocardial infarction and stroke [28]. Neurodegenerative diseases such as Alzheimer’s are associated with neuroinflammation, where inflammatory responses may damage neurons, leading to cognitive decline [29].

Macrophages play a pivotal role in inflammation and tissue repair. Tissue-resident macrophages are key regulators of tissue healing, renewal and scar formation. During the early stages of injury, macrophages serve as major sources of chemokines and TNF-α, initiating the inflammatory response. As inflammation subsides, macrophages adopt a healing phenotype. They secrete PDGF, TGF-β, IGF-1 and VEGF-A. These factors drive cell proliferation and angiogenesis. Additionally, anti-inflammatory macrophages activated by IL-10 release anti-inflammatory substances like IL-10 and TGF-β, playing a crucial role in suppressing immune responses. Therefore, precise regulation of macrophage phenotypes at different stages of tissue repair is essential for coordinating inflammation and promoting wound healing [30].

Inflammation exhibits high adaptability, adjusting to various stressors. Parainflammation, a low-grade inflammatory state between homeostasis and overt inflammation, is an intermediate state between baseline homeostasis and classical inflammation, primarily driven by tissue-resident macrophages to help tissues adapt to stress. However, if the stress persists or intensifies, parainflammation may transition into chronic inflammation. Long-term psychological stress and unhealthy lifestyles can place the body in a state of chronic low-grade inflammation, which, if unaddressed, may lead to a range of chronic diseases [31]. In chronic diseases, inflammation disrupts normal tissue structure and function. It promotes fibrosis, abnormal proliferation and tissue remodeling. These changes drive disease progression.

With aging, the immune system gradually declines and chronic low-grade inflammation, or ‘inflammaging’ becomes more prevalent. This inflammatory state accelerates biological aging, leading to functional decline in organs and systems [32]. Inflammaging is closely associated with age-related diseases such as Alzheimer’s disease [33], cardiovascular diseases [34] and type 2 diabetes [35]. Besides, genome-wide studies have provided important insights into the relationship between inflammation and genetics. For example, polymorphisms in the C-reactive protein (CRP) gene are closely associated with the risk of chronic inflammatory diseases. Certain CRP variants may lead to elevated expression levels, increasing an individual’s susceptibility to cardiovascular and autoimmune diseases [36]. These findings offer new avenues for early diagnosis and personalized treatment.

In summary, inflammation plays a complex and critical role in both physiological and pathological processes. Its dysregulation can contribute to the onset and progression of numerous chronic diseases. Further work on inflammatory mechanisms and gene–environment interactions should inform new therapies. This may ultimately improve patient care.

### The phases of inflammation

Inflammation is a dynamic and tightly regulated biological process that occurs in response to tissue injury, infection or other harmful stimuli [37]. Inflammation has three interconnected phases: initiation, resolution and chronic inflammation. Each phase involves distinct molecular and cellular events. Together they maintain tissue homeostasis. Dysregulation contributes to disease.

The initiation phase is marked by the rapid activation of innate immune responses. Upon injury or infection, sentinel cells detect danger signals. Tissue-resident macrophages and DCs release cytokines and chemokines. These recruit neutrophils and monocytes to the site. Neutrophils act as first responders. They traverse the vascular endothelium and infiltrate inflamed tissue. There, they phagocytose pathogens and cellular debris. They also release reactive oxygen species (ROS), proteolytic enzymes and antimicrobial peptides [38]. These actions, though essential for pathogen clearance, may exacerbate tissue injury. In parallel, acute-phase proteins such as CRP are systemically upregulated [39]. These proteins act not only as biomarkers of inflammation but also contribute to innate immune responses by activating the complement system and facilitating opsonization. As the local immune response amplifies, mechanisms governing tissue repair are simultaneously initiated.

Transitioning from inflammation to restoration requires the tightly controlled resolution phase. This phase is not merely a passive cessation of inflammation but an active process involving phenotypic reprogramming of immune cells and biosynthesis of specialized pro-resolving mediators (SPMs). Macrophages play a central role in this transition, undergoing a functional switch from the pro-inflammatory M1 phenotype to the anti-inflammatory and reparative M2 phenotype. This shift is regulated by cytokines such as IL-4 and IL-10 and accompanied by a reduction in pro-inflammatory cytokine production [40]. SPMs, including resolvins, protectins and maresins, facilitate the clearance of apoptotic cells, microbial remnants and necrotic debris via efferocytosis. Moreover, they inhibit further neutrophil recruitment and promote macrophage-driven tissue remodeling, thus restoring homeostasis. Failure to resolve inflammation may lead to its chronic persistence. This state involves sustained infiltration of immune cells. It also involves ongoing release of inflammatory mediators. Progressive tissue damage, fibrosis or loss of function may ensue [41].

Chronic inflammation is increasingly recognized as a driving factor in a range of pathological conditions, including rheumatoid arthritis [42], type 2 diabetes [43], cardiovascular diseases [44] and cancer [45]. In this persistent inflammatory state, the immune response remains aberrantly activated, often with a distorted balance between pro- and anti-inflammatory signals. Neutrophils, which dominate the early acute phase, can cause collateral damage when excessively activated or inadequately cleared [46]. Meanwhile, macrophages remain functionally plastic, exerting either detrimental or beneficial roles depending on their microenvironmental cues [47]. The local cytokine milieu shapes cell phenotypes and functions. Key factors include IFN-γ, TGF-β and CXCL12 [48–50]. This milieu determines inflammatory outcomes.

Inflammation comprises a series of highly coordinated and stage-specific responses that collectively aim to protect and repair tissues. Each phase plays an indispensable role in restoring homeostasis. However, when resolution fails, inflammation becomes chronic, fueling disease pathogenesis. Understanding how inflammation resolves is critical for therapy development. Key questions center on immune cell function and mediator activity. Precision approaches require this mechanistic insight [51].

## The overview of bacterial extracellular vesicles

BEVs are bilayered membranous structures (20–400 nm). Both Gram-negative and Gram-positive bacteria secrete them. Biogenesis is triggered by membrane instability or stressors. Common triggers include antibiotics, pH shifts and temperature changes. As illustrated in Figure 1, BEVs primarily consist of outer membrane proteins, lipopolysaccharides (LPS), phospholipids and occasionally periplasmic proteins, cytoplasmic proteins, DNA and RNA. These vesicles are crucial in immune activation, carrying bacterial antigens, making them promising candidates for nonreplicating vaccines. BEVs also contain bactericidal components like murein hydrolases, which degrade bacterial peptidoglycan, presenting a potential therapeutic approach for infections. Moreover, BEVs are involved in bacterial defense, gene transfer and intercellular communication, serving as vehicles for genetic exchange and virulence factors. Overall, BEVs are critical to bacterial survival, pathogenesis and therapeutic applications, including vaccine development and infection treatment [52]. Recent studies have significantly expanded our understanding of BEV functions. For instance, gut microbiota-derived outer membrane vesicles (OMVs) have been shown to regulate intestinal homeostasis [53], and *Fusobacterium nucleatum*-derived OMVs can aggravate diseases like rheumatoid arthritis [54] and promote oral cancer metastasis by activating autophagy [55]. Furthermore, OMVs from pathogens like Helicobacter pylori have been implicated in extrapolar diseases such as Alzheimer’s disease via C3-C3aR signaling [33].

**Figure 1:**
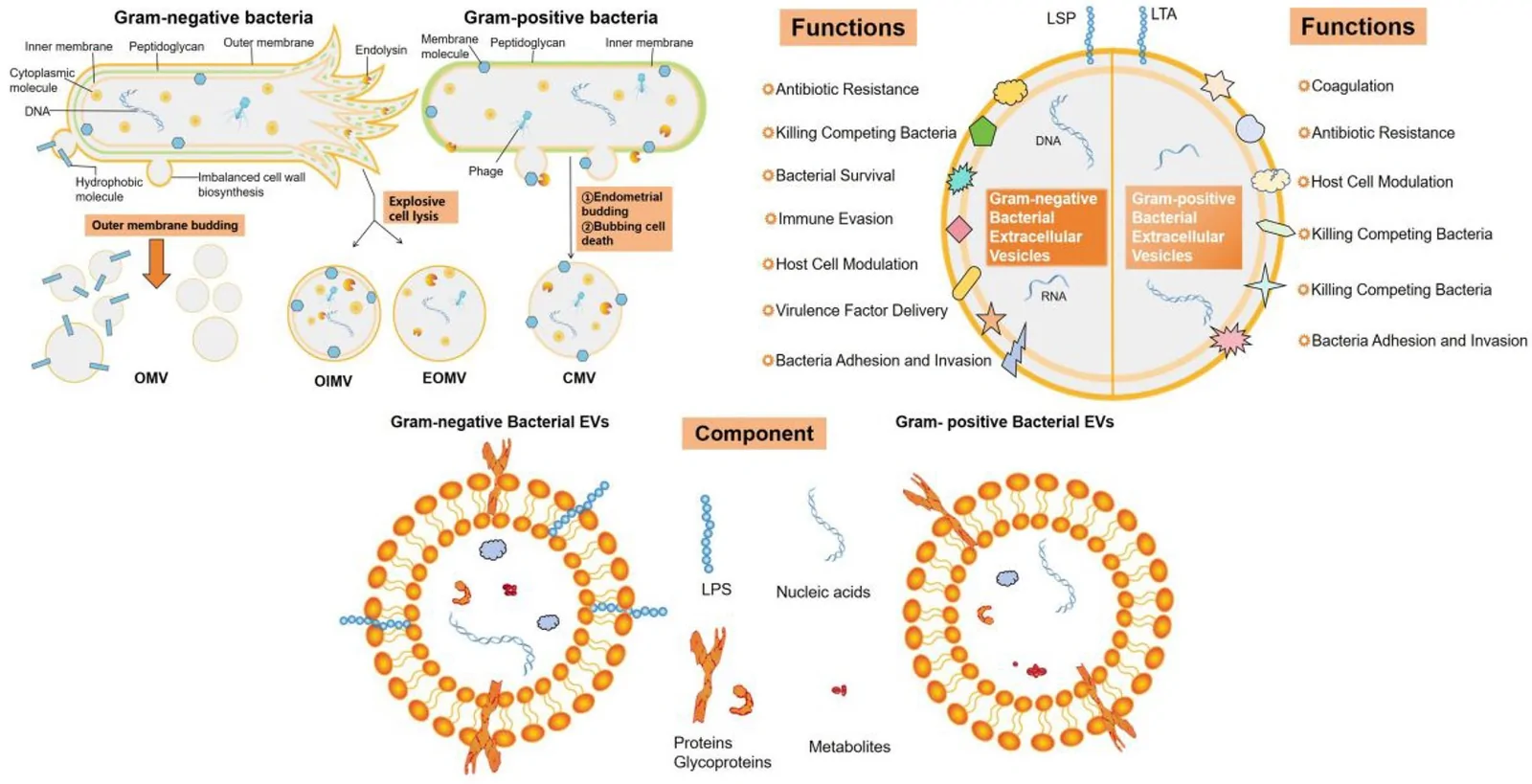
Overview of BEVs in Gram-negative and Gram-positive bacteria. The structure, function and composition of EVs in Gram-negative and Gram-positive bacteria are significantly different. The left figure shows the mechanism of EV formation in Gram-negative bacteria, where OMVs are formed by budding from the outer membrane, and in Gram-positive bacteria, cell membrane vesicles (CMV) are produced by budding or explosive cell lysis of the cytoplasmic membrane. The right figure outlines the key functions of BEVs, including antibiotic resistance, bacterial survival, immune escape and bacterial adhesion. The following figure provides a detailed introduction to the components of Gram-negative and Gram-positive BEVs, such as LPS, proteins, nucleic acids and metabolites, which play various roles in bacterial pathogenesis and interactions with host cells. This original figure was designed by the authors.

### The origin and classification of BEVs

BEVs originate from Gram-negative and Gram-positive bacteria and are produced through distinct biogenetic pathways such as outer membrane blebbing or explosive cell lysis. BEVs are classified into several subtypes based on their structure and formation mechanism.

#### Outer membrane vesicles

OMVs, which are spherical, are the best-characterized BEVs, mainly produced by Gram-negative bacteria via outer membrane blebbing. They typically range from 20 to 300 nm in diameter and possess a lipid bilayer structure derived from the outer membrane. OMVs carry a diverse array of bioactive molecules. These include outer membrane proteins, LPS, phospholipids and lipoproteins. They also carry flagellin, periplasmic proteins and cytoplasmic proteins. DNA and RNA are also present [56]. OMVs have garnered significant interest as vaccine candidates due to their inherent immunogenicity and safety profile. For instance, engineered OMVs from *Acinetobacter baumannii* exhibit superior homogeneity, protein yield and reduced toxicity compared to native OMVs. Immunization with low-endotoxin outer membrane vesicles provides robust protection in murine models of pneumonia and bacteremia, underscoring their potential in vaccine development. Beyond vaccines, OMVs act as natural delivery vehicles, capable of targeting host tissues and modulating immune responses. A notable example is OMVs from *Pseudomonas aeruginosa*, which traverse airway mucus to deliver regulatory small RNAs (sRNAs) to epithelial cells. These sRNAs can suppress LPS-induced IL-8 production by interfering with MAP kinase signaling, highlighting a novel cross-kingdom communication mechanism that contributes to immune evasion and chronic infection. OMVs usually trap periplasmic components and are implicated in many bacterial processes, such as biofilm formation, antibiotic resistance and nucleic acid transfer. Recent work shows that OMVs can also induce epithelial cell mitophagy to enhance intracellular survival of pathogens like Neisseria gonorrhoeae in a PorB-dependent manner [57, 58] and that sub-inhibitory antibiotics can affect microbial processes like ferrihydrite reduction via OMVs [59]. During infection, host cells absorb OMVs through a variety of different routes, including receptor-mediated endocytosis and membrane fusion. Host cell responses to OMV uptake have been characterized in different cell types and range from the initiation of a proinflammatory response in immune cells to an immunomodulatory effect in nonimmune cells. For example, H. pylori OMVs can induce astrocyte reactivity and neuronal damage *in vivo* [60, 61], while gut pathobiont-derived OMVs can drive liver inflammation and fibrosis [62]. Conversely, probiotic-derived OMVs from *Escherichia coli* O83 or *Parabacteroides goldsteinii* can exhibit anti-inflammatory effects and prevent allergy or suppress skin inflammation [63, 64].

#### Cytoplasmic membrane vesicles

Cytoplasmic membrane vesicles (CMVs) are typically generated by Gram-positive bacteria following explosive cell lysis, though similar structures have also been observed in Gram-negative species [65]. Ranging from 20 to 400 nm, CMVs encapsulate a broader spectrum of cytoplasmic contents, including peptidoglycan fragments, nucleic acids, cytoplasmic proteins and metabolites [65, 66]. These vesicles function in horizontal gene transfer, delivering genetic material between cells to promote genomic plasticity and adaptive evolution [67]. CMVs also play pivotal roles in virulence, serving as carriers for toxins and immune modulators that disrupt host defense systems. Additionally, they contribute to biofilm development and maintenance, enhancing bacterial survival under stress [68]. The ability of CMVs to induce or dampen immune responses makes them key players in microbial pathogenesis and host interaction [52].

#### Outer-inner membrane vesicles and explosive outer membrane vesicles

Outer-inner membrane vesicles (OIMVs) and explosive outer membrane vesicles (EOMVs) arise from explosive lysis events in Gram-negative bacteria, resulting in complex vesicles that may encapsulate both inner and outer membrane components, as well as cytoplasmic material. OIMVs exhibit a distinctive double-membrane structure with an interleaving peptidoglycan layer, which sets them apart from traditional OMVs. Due to their lytic origin, OIMVs and EOMVs often contain a more random and heterogeneous mix of cargo, including debris from disrupted cells. These vesicles contribute to interbacterial communication, horizontal gene transfer, biofilm formation and immune modulation. For example, they can mediate the dissemination of antibiotic resistance genes or subvert host immunity, thereby promoting bacterial persistence and multidrug resistance. The biogenesis of these complex vesicles can be regulated by environmental cues such as pH, as a pH-sensitive motif in an outer membrane protein has been shown to activate vesicle production [69, 70].

#### Tubular structures and membranes

Tubular structures and membranes (TSMs) represent another form of membrane-derived structure, observed in both Gram-positive and Gram-negative bacteria. These tubular protrusions, formed from cytoplasmic or outer membranes, can extend between cells to create bridges for molecular trafficking. For instance, *Staphylococcus aureus* produces interconnected OMV chains and TSMs, facilitating the intercellular transfer of membrane proteins. TSMs participate in biofilm formation, immune regulation and toxin delivery and may also play roles in intracellular signaling and apoptosis. These structures underline the architectural versatility of bacterial membranes in forming communication and defense networks.

The origin of BEVs is intricately tied to bacterial growth dynamics and stress adaptation, while their classification reflects the diversity in structure, biogenesis and function. From OMVs to CMVs, OIMVs/EOMVs and TSMs, each BEV subtype contributes uniquely to bacterial survival, communication and pathogenesis. BEV biogenesis and function inform bacterial physiology. These insights also enable vaccine development, drug delivery and diagnostics.

### The structure and composition of BEVs

BEVs, particularly OMVs derived from Gram-negative bacteria, are nanoscale, bilayered structures that play crucial roles in intercellular communication, pathogenesis and adaptation to external environments [71]. These vesicles typically range from 20 to 400 nm in diameter [72], with their size and morphology varying depending on bacterial species and environmental conditions. OMVs have a characteristic double-membrane structure derived from the outer membrane of their parent cells, providing stability and the ability to encapsulate and protect various biomolecules. BEV morphology varies widely. Forms include spherical, elliptical, granular and spindle-like shapes. This diversity reflects heterogeneous formation pathways [73].

The composition of BEVs is highly diverse and intricately tied to their biological functions. Their lipid bilayers are primarily composed of phospholipids and lipoproteins, which are essential for maintaining membrane integrity and mediating interactions with host cells. The lipoprotein N-acyltransferase in Bacteroides has been characterized, highlighting the importance of lipoproteins in OMVs [74]. In Gram-negative bacteria, the outer membrane also contains LPS, potent immunostimulatory molecules critical for host-pathogen interactions. The structural features of LPS, particularly the O-antigen, determine how OMVs interact with and enter host cells. OMVs from pathogenic strains, which are rich in LPS, are typically associated with high immunogenicity and virulence. Lipid A in OMVs has been shown to shield bacteria from antimicrobial peptides like polymyxins [75], and OMVs contribute to *P. aeruginosa* resistance to antimicrobial peptides in acidic environments [76]. Recent advances have enabled the engineering of low-endotoxin vesicles by modifying LPS content, thus improving their safety for therapeutic applications. Moreover, the lipid composition of BEVs is adaptable, allowing for changes in response to environmental cues, enhancing vesicle fusion and targeting efficiency, which holds significant potential for drug delivery applications.

Proteins are another key functional component of BEVs. These include outer membrane proteins, periplasmic and cytoplasmic proteins and virulence factors that vary by bacterial species. For instance, Omp38 of carbapenem-resistant *A. baumannii* mediates mtDNA release to activate the cGAS-STING pathway and induce inflammation [77], and hemagglutinin protease HapA associated with *Vibrio cholerae* OMVs disrupts epithelial junctions [78]. In Gram-positive BEVs, lipoproteins and iron-binding proteins are often enriched, and these proteins are known to stimulate TLR2-mediated immune responses [79]. Pathogenic bacteria often incorporate cytotoxic proteins into their vesicles. For example, *Bacillus anthracis*-derived BEVs contain factors lethal to macrophages, while *Porphyromonas gingivalis* releases LPS-rich vesicles that disrupt epithelial barriers [80]. Recent studies show *P. gingivalis* OMVs can impede bone regeneration by inducing ferroptosis [81]. In *S. aureus*, vesicular proteins are implicated in antibiotic resistance, bacterial communication and the regulation of further EV production. These protein cargos are not only essential for host–pathogen interactions but also play crucial roles in bacterial survival, adaptation and immune evasion.

In addition to lipids and proteins, BEVs also contain nucleic acids, including DNA and various forms of RNA. These nucleic acids contribute to horizontal gene transfer, interbacterial communication and, in some cases, host gene regulation. Certain small RNAs carried by OMVs can modulate host immune responses or influence bacterial virulence gene expression, expanding the functional role of BEVs beyond structural or enzymatic activities [13]. sRNA52320 from *P. aeruginosa* is a classic example [82], and recent work shows sRNA-enriched OMVs from *Vibrio alginolyticus* are decisive architects of biofilm assembly [83].

BEVs also frequently contain other biologically active molecules, such as metabolites, polysaccharides and peptidoglycan fragments. Peptidoglycan fragments, particularly in vesicles derived from Gram-positive bacteria, can activate innate immune receptors. The presence of secondary metabolites and signaling molecules further underscores the dynamic role of BEVs in bacterial adaptation and survival. Human gut bacteria tailor OMV cargo for the breakdown of diet- and host-derived glycans [61], and Bacteroides sphingolipids in OMVs can promote anti-inflammatory responses through the mevalonate pathway [84].

Importantly, the structure and composition of BEVs are not static but are modulated by bacterial species, environmental conditions and physiological states. Pathogenic bacteria may enrich their vesicles with specific virulence factors or antigens to promote infection, while probiotic strains may secrete BEVs with immunoregulatory or protective properties. This compositional plasticity not only enhances bacterial fitness in diverse environments but also broadens the functional scope of BEVs in microbial ecosystems. OMVs, in particular, facilitate material exchange, mediate microbial competition or cooperation and participate in biofilm formation. These attributes highlight the growing recognition of BEVs as essential players in microbial biology, as well as promising candidates for biomedical applications such as vaccines, targeted drug delivery and diagnostic biomarkers. Differential temporal release and lipoprotein loading in *B. thetaiotaomicron* BEVs [85] and the profiling of LTA/LPS-positive EVs in plasma as biomarkers for infection [86] are examples of this plasticity.

However, a critical issue remains. Across BEV studies, authors frequently use ‘OMV’ as a generic label for Gram-negative vesicle preparations even though multiple BEV subtypes can co-occur, are similar in size, and therefore copurify. This leads to misattribution of cargo and downstream functions to the wrong subtype. Multiple reviews explicitly note that BEV populations are heterogeneous; that there are no universal markers; that physical-property-based isolation is insufficient; and that copurification leads to contradictory cargo and functional claims [87, 88]. Juodeikis and Carding [87] highlight that DNA is often ‘perceived’ in OMV preparations but has been unequivocally confirmed in other BEV types and that many functional studies do not distinguish vesicle subtypes. In parallel, standardization failures in quantification can reverse immunogenicity conclusions. Bitto *et al*. [89] show that protein-based normalization can mask strain differences, whereas particle-number normalization reveals differences in TLR/NF-κB/IL-8 responses and in DNA/RNA cargo. Although we have some available classification principles, they often lack credibility in the articles. Therefore, we believe that to enhance the credibility of BEV classification across different studies, ensure cross-laboratory applicability and avoid circular definitions, a minimum set of evidence packages should be provided before assigning subtype labels. This includes electron microscopy to distinguish single‑versus double‑bilayer morphology [87, 90]; assessment of outer versus inner membrane marker enrichment to determine whether inner‑membrane proteins are present or the preparation primarily contains outer membrane and periplasmic proteins (characteristic of OMVs) [91, 92]; DNase protection assays to ascertain whether DNA is intraluminally protected or arises from external contamination [90, 93]; LPS quantification combined with Gram‑status markers to predict LPS‑driven immunogenicity and avoid conflation between Gram-negative and Gram‑positive vesicles [94, 95]; and dose normalization based on particle number rather than protein mass, which prevents contradictory immunogenicity conclusions [89].

Overall, BEVs are structurally complex and compositionally rich entities that reflect the unique biology of their parent organisms. Their ability to encapsulate diverse biomolecules and engage in intricate interactions with host cells positions them at the forefront of microbial communication and pathogenesis. With ongoing advancements in vesicle isolation, characterization and engineering, BEVs are poised to play an increasingly pivotal role in both fundamental microbiology and translational medicine.

### Separation and modification

The isolation and modification of BEVs are essential steps in advancing bacteriological research and biomedical applications. Efficient separation methods are fundamental to obtaining high-purity BEVs for downstream use. Among these, differential centrifugation remains a widely used approach, where biological samples such as bacterial culture supernatants are first preprocessed to remove large debris. Low-speed centrifugation eliminates cell fragments, followed by higher-speed centrifugation steps to isolate vesicles based on their sedimentation coefficients. Although this method is simple and accessible, its yield and purity are heavily influenced by centrifugation parameters, and excessive centrifugal force may damage vesicle integrity [96].

To improve purity, density gradient centrifugation separates components according to buoyant density using gradient solutions like sucrose or iodixanol. BEVs, particularly OMVs, typically migrate to lighter fractions due to their lower density compared to proteins, flagella or pili. While this method offers higher purity, it is more time-consuming and technically demanding [97]. Ultrafiltration, employing membranes with defined pore sizes, enables size-based separation and is suitable for large-scale processing. However, issues such as membrane clogging and vesicle deformation from shear stress may occur [12]. Alternatively, size exclusion chromatography (SEC), also known as gel filtration, uses porous beads to sort particles by size. Larger particles elute first, while smaller ones are retained within the pores and elute later. SEC is effective for achieving high-resolution separation and is ideal for removing small contaminants such as lipoproteins, thereby improving BEV purity.

For applications requiring selective isolation, immunomagnetic separation offers a targeted approach using BEV-specific antibodies bound to magnetic beads. Although highly specific and useful for small, complex samples, it relies on the availability of high-affinity antibodies and may introduce interference from the magnetic materials [98]. Innovative methods continue to emerge, such as fluorescence-activatable nanoprobes for anaerobic tracking of gut microbiota-derived vesicles [99] and specific labeling of OMVs with antibiotic-conjugated probes for early infection detection [100]. Overall, each method presents distinct advantages and limitations, and the choice depends on the experimental objectives, sample characteristics and processing scale. Continued innovation in separation technologies is vital for optimizing BEV isolation and supporting their expanding roles in research and therapy.

Following isolation, BEV modification serves as a key biotechnological strategy to enhance their functionality, targeting ability and biocompatibility for diverse biomedical applications [52]. One approach involves parental strain engineering, where bacterial strains are genetically modified to produce BEVs containing specific therapeutic molecules, such as proteins, enzymes or antibodies [101, 102]. Complementing this, post-isolation engineering allows for physical or chemical modifications after vesicle extraction, such as lipid fusion for drug loading or surface conjugation for targeted delivery [12]. To further improve specificity, targeting enhancement strategies incorporate molecules like antibodies or receptor ligands onto BEVs, enabling precise delivery to diseased tissues such as tumors [103]. Additionally, surface modifications such as PEGylation can significantly reduce immunogenicity and cytotoxicity, improving systemic biocompatibility [104]. BEVs can also be loaded with therapeutic cargo, including nucleic acids, chemotherapeutics or anti-inflammatory agents, making them promising vehicles for gene therapy, cancer treatment [105] and immune modulation.

However, the success of these modifications hinges on the ability to precisely control the type, quantity and localization of functional molecules [106]. Ensuring the safety, stability and compatibility of engineered BEVs is paramount [107]. Advances in genetic engineering, nanotechnology and biomaterials science continue to refine these strategies, unlocking new potential for BEVs in precision medicine [107]. Rationally designed BEV modification not only enhances their therapeutic utility but also opens new frontiers for their application in targeted therapy, diagnostics and immunotherapy [106].

Engineered BEVs show potential in vaccines, drug delivery and cancer immunotherapy. However, OMVs carry pathogen-associated molecular patterns. These interact with innate immune receptors and trigger responses. Therefore, during the engineering process, the potential changes they may introduce must be carefully considered. Lipid A, the endotoxic component of LPS, is the primary TLR4 ligand that drives inflammatory responses [89]. Wild-type hexa-acylated lipid A can elicit acute and even lethal inflammation; thus, reducing its TLR4 agonistic activity has been a central aim of OMV engineering. Targeted deletions of late-stage acyltransferase genes alter lipid A acylation, converting hexa-acylated forms to penta- or tetra-acylated variants [106, 108]. *In vitro* studies using HEK293 cells expressing human TLR4 demonstrated that penta-acylated lipid A from ΔmsbB or ΔhtrB strains induces 10- to 60 000-fold reductions in NF-κB activation compared with wild-type lipid A, and monocyte activation tests have shown 300- to 800-fold reductions in IL-6 release depending on the strain and modification. Notably, penta- and tetra-acylated lipid A are very poor activators of human TLR4 [108]. Chemical extraction with detergents removes LPS and lipoproteins, partially reducing both TLR4 and TLR2 activation [106, 109]. However, this process also depletes protective antigens and TLR agonists that provide inherent adjuvanticity, can compromise vesicle stability and aggregation propensity, and introduces manufacturing and purification challenges [66, 109].

## The role and mechanism of BEVs in inflammatory regulation

EVs secreted by innate immune cells and bacteria play pivotal roles in regulating inflammation. Inflammatory cells, such as macrophages, secrete EVs that carry bioactive molecules, including lipids, proteins and miRNAs, to influence immune responses. For instance, macrophage-derived EVs promote osteogenesis and tissue regeneration in periodontitis by modulating M1/M2 macrophage polarization. In contrast, EVs from pro-inflammatory macrophages exacerbate alveolar bone loss by inducing cytokine release and disrupting bone metabolism [110]. As shown in Figure 2A, bacteroides acidifaciens and its derived extracellular vesicles improve dextran sulfate sodium (DSS)-induced colitis. Figure 2B illustrates how M2 macrophage-derived EVs can contribute to immune reprogramming, reversion of cell states and periodontal protection [111]. Figure 2C summarizes the results and conclusion of the effects of nonalcoholic steatohepatitis (NASH) fEVs (fecal-derived extracellular vesicles). fEVs contribute to intestinal barrier dysfunction. They carry lipopolysaccharide and act via the non-muscle myosin light-chain kinase (nmMLCK) pathway [112]. This mediates downstream liver injury. Figure 2D highlights how BEVs interact with host cells via PRRs, triggering inflammatory cytokine production and exacerbating tissue damage [111]. The dual roles of bacteria-derived EVs acting both as mediators of inflammation and as potential therapeutic agents open up a broad range of applications for EV-based therapies. However, these vesicles also introduce complexities that require careful consideration [113]. For instance, Hong *et al*. [54] observed that the hemoglobin-like cell nucleus of *F. nucleatum* was enriched in patients with rheumatoid arthritis and was positively correlated with the severity of rheumatoid arthritis. *F. nucleatum* also exacerbated arthritis in mice with collagen-induced arthritis (CIA). The outer membrane vesicles (OMVs) of *F. nucleatum* containing the pathogenic determinant FadA migrated to the joints and triggered a local inflammatory response. FadA acted on synovial macrophages, activating the Rab5a GTPase involved in vesicle transport and inflammatory pathways, as well as the key regulatory factor YB-1 of inflammatory mediators. While they may serve as promising vaccine candidates or therapeutic agents to modulate immune responses, their pro-inflammatory properties, particularly in chronic diseases, must be controlled to avoid exacerbating tissue damage [114]. Understanding the delicate balance between the beneficial and harmful effects of EVs is essential for their effective application in clinical settings [115], highlighting the need for targeted approaches that can modulate their inflammatory impact without compromising their therapeutic potential. It is worth noting that among various periodontal pathogens, OMVs derived from *F. nucleatum* play a unique and crucial role in the occurrence and development of periodontitis, and their independent pathogenicity and specific mechanism have been confirmed by the latest research [116, 117].

**Figure 2:**
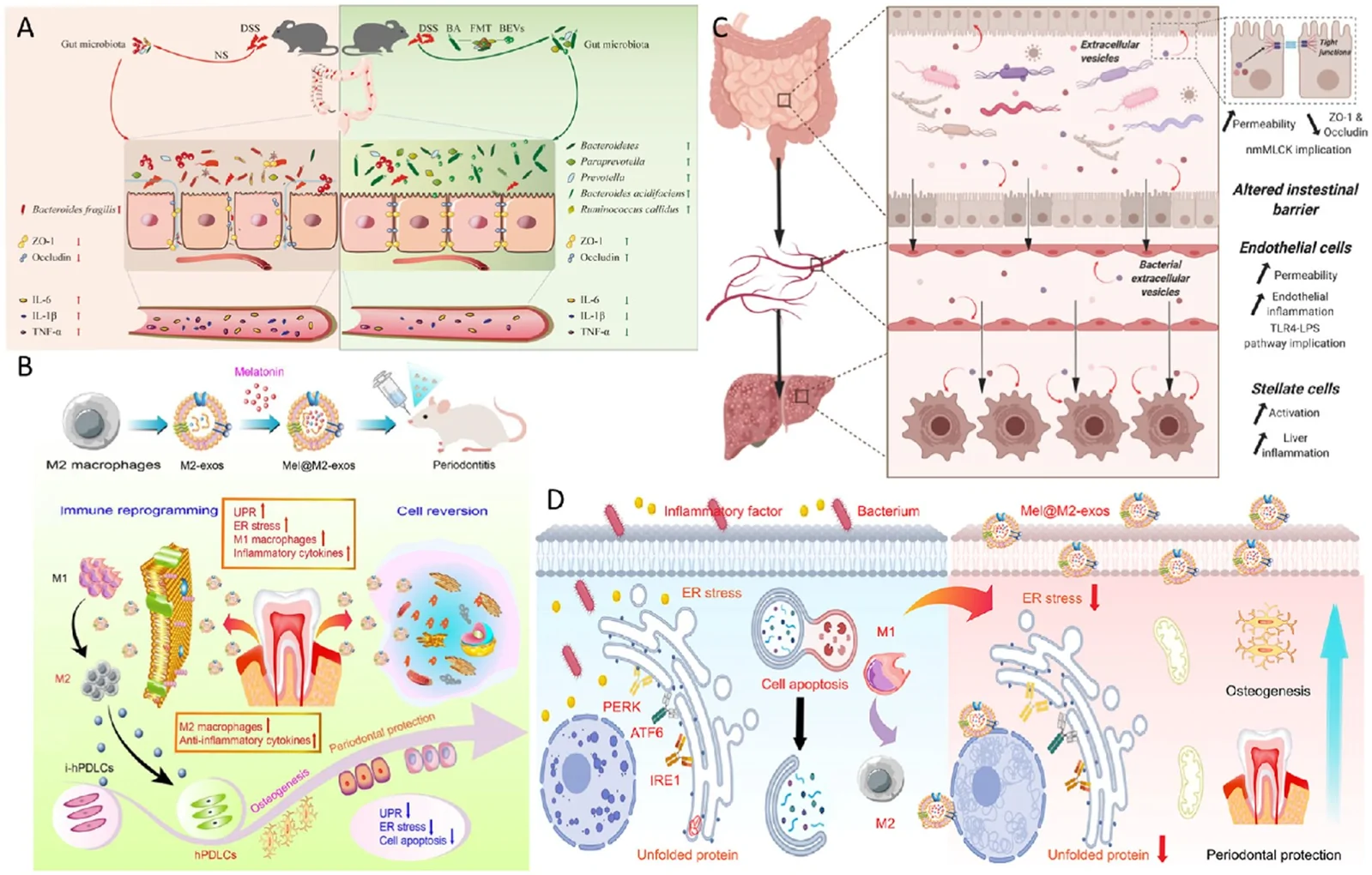
Mechanisms of BEVs in inflammatory diseases: (**A**) the impact of gut microbiota and BEVs on the intestinal barrier. Reprinted with permission [110]. Copyright 2023, The Author(s). (**B**) Schematic representation of engineered M2 macrophage-derived BEVs for treating inflammatory bone loss in periodontitis through mediating ER stress and immune reprogramming. Reprinted with permission [111]. Copyright 2023, The Author(s). (**C**) Schematic illustration of the interaction between the intestinal barrier, endothelial cells and stellate cells in the context of gut—liver axis disruption. Reprinted with permission [112]. Copyright 2023, The Author(s). (**D**) Schematic diagram of engineered M2 macrophage-derived EVs inhibiting ER stress and cell apoptosis for periodontitis treatment. Reprinted with permission [111]. Copyright 2023, The Author(s).

### Anti-inflammatory roles and mechanisms of BEVs

Recent studies show that OMVs from *P. gingivalis* and *Aggregatibacter actinomycetemcomitans* activate host cell inflammatory factor release, while M1 macrophage-derived EVs may contribute to periodontitis-associated bone loss. Conversely, M2 macrophage and dendritic cell EVs promote bone regeneration in animal models. Although bacterial EV applications in periodontal therapy are nascent, *P. gingivalis* OMVs show promise as vaccine candidates [118]. Periodontitis, a chronic inflammatory disease involving innate and adaptive immunity, is closely tied to immune cell-derived EVs. Modulating these EVs to alter cell behavior and differentiation may effectively combat periodontitis-related bone loss. In experimental periodontitis models, M1 and M2 macrophage EVs inhibited and promoted osteogenesis, respectively [16]. *In vitro*, M1 EVs suppressed mesenchymal stem cell (MSC) osteogenic differentiation, while M2 EVs enhanced mineralization. *In vivo*, preloading M1 EVs with miR-303-5p inhibitors significantly reduced alveolar bone loss [111, 119]. The anti-inflammatory roles and mechanisms of bacterial and immune cell-derived EVs present a promising frontier in periodontal therapy. As periodontitis involves complex interactions between innate and adaptive immunity, EVs offer a strategic approach to mitigating inflammation and promoting osteogenesis, particularly those from M2 macrophages and DCs. Experimental evidence confirms that M1 macrophage EVs exacerbate bone loss, whereas M2 EVs enhance mineralization and tissue repair, especially when engineered to suppress pro-inflammatory miRNAs like miR-303-5p [119, 120]. Chen *et al*. [121] found that M2 EVs promoted bone marrow MSC osteogenesis via multiple miRNAs and IL-10/IL-10R signaling, reducing bone resorption in periodontitis mice. Similarly, Cui *et al*. [111] reported melatonin-loaded M2 EVs enhanced osteogenic/cementogenic potential of human periodontal ligament stem cells (hPDLSCs) under inflammation. These EVs also drove M1-to-M2 reprogramming, mitigating inflammation and accelerating healing (Table 1).

**Table 1:** Classification of anti-inflammatory studies of probiotic-derived BEVs by strength of evidence.

Producer BEV	Study/model	Anti-inflammatory outcome	Mechanistic test type	Evidence strength category	Notes distinguishing causal evidence vs correlative conclusion
*Bacteroides fragilis* OMVs (PSA^+^)	TNBS colitis mice; DC transfer experiments	↓Weight loss/colon shortening/histology; ↓TNF-α/IL-17; ↑IL-10; Treg induction	Bacterial mutant (ΔPSA lost protection); host DC-intrinsic Gadd45a required; host ATG16L1 (DC-specific) and NOD2 required; TLR2 recognition on DCs	A	Causal: loss-of-function in producer (ΔPSA) and host requirements (Gadd45a, ATG16L1, NOD2) demonstrated *in vivo*; DC/TLR2 mechanism defined [122, 123]
*Escherichia coli* Nissle 1917 OMVs	DSS colitis mice (oral OMVs pretreatment)	↓Weight loss; improved clinical/histology; TJ changes (↑ZO-1/claudin-14, ↓claudin-2)	Pathway/barrier markers; no host KO; no bacterial mutant	B	Correlative: barrier/cytokine markers support efficacy; no genetic or receptor KO evidence [124]
*Lactobacillus paracasei* EVs	DSS colitis mice; HT29 LPS model	↓IL-1α/IL-1β/IL-2/TNF-α; ↑IL-10/TGFβ; ↓NF-κB/iNOS/COX-2; improved DAI/colon length	*In vitro* CHOP siRNA; ER-stress inhibitors; No *in vivo* KO	B	Mechanism inferred mainly from *in vitro* perturbations; *in vivo* lacks genetic validation [125]
*Lactobacillus kefirgranum* PRCC-1301 EVs	DSS acute; chronic colitis in IL-10 mice; Caco-2 assays	Attenuated weight loss/colon shortening; restored TJ (ZO-1/claudin-1/occludin); ↓p–NF-κB p65/IκBα	Pathway/barrier markers; IL-10 used as disease model; no receptor KO	B	Correlative: NF-κB/TJ marker changes; IL-10 denotes severity model, not pathway necessity [126]
*Akkermansia muciniphila* EVs	DSS colitis mice; RAW264.7 LPS model	Prevented colitis; ↓injury; ↑MUC2; ↓permeability; ↓NO/TNF-α/IL-1β *in vitro*; microbiota shifts	EV uptake shown; barrier/permeability and microbiota changes; no KO	B	Correlative: barrier/microbiota associations without genetic proof [127]
*Bifidobacterium longum* NSP001 EVs (NEVs)	DSS colitis mice; biodistribution; pseudo-germ-free	Improved barrier; modulated T cell differentiation; ↑SCFAs; STAT3 suppression; effect persists in pseudo-germ-free	Pseudo-germ-free dependence test; pathway markers; no host KO or producer mutant	B	Correlative: suggests microbiota-independent component but no genetic loss-of-function [127]
*Bacteroides thetaiotaomicron* BEVs	DSS colitis mice; BMDM/THP-1	↑IL-10; improved survival/weight/DAI; epigenetic reprogramming (↑H3K4me1)	TLR2 mediation shown in THP-1; trained-immunity marks; no *in vivo* KO	B	Correlative: receptor involvement inferred *in vitro*; no *in vivo* knockout [128]
*Lactobacillus rhamnosus* GG BEVs	Polymicrobial sepsis (FIP); macrophage phagocytosis assays	↑Survival; ↑bacterial clearance; ↓serum cytokines; ↓organ injury	Pharmacologic antagonist (Boc2 for FPR1/2) *in vitro*; no *in vivo* receptor KO	B	Correlative *in vivo*; partial causal support from *in vitro* receptor blockade [129]
*Propionibacterium freudenreichii* EVs	HT-29 *in vitro* inflammation model	↓NF-κB activation; ↓IL-8-dependent)	EV surface proteolysis test; partial dependence on SlpB inferred; no animal model	C	*In vitro* only; effector implication (SlpB) via perturbation [130]
*Propionibacterium freudenreichii* EVs	HT-29 *in vitro*; media comparison (UF vs YEL)	UF-EVs more abundant/smaller; stronger ↓NF-κB/IL-8	Bacterial mutant (ΔslpB EVs reduced effect); cargo shifts by culture conditions; no animal model	C	*In vitro* only; producer-factor dependence shown without *in vivo* testing [131]
*Lactobacillus paracasei* EVs (skin)	Human dermal fibroblasts; 3D skin equivalent (TNF-α)	↓MMP1/IL-6/IL-8; ↑collagen; restored epidermal morphology	Uptake/antioxidant capacity; no inhibitors/KO	C	*In vitro* only; topical anti-inflammatory phenotype [132]
*Akkermansia muciniphila* EVs	Caco-2 LPS-induced inflammation	↓ROS/NO/MDA; ↑CAT; ↓TNF-α/IL-1β/IL-6; MAPK/MyD88/TRIF modulation; ↑TGFBR2	Transcriptomics/WB pathway markers; no KO	C	*In vitro* only; pathway modulation without functional genetic tests [133]

Classification of probiotic/commensal-derived BEV studies into evidence categories A–C, noting *in vivo* anti-inflammatory outcomes and whether causal mechanisms were proven with host knockouts or bacterial mutants. This helps distinguish mechanistic causality from correlative pathway readouts across major BEV reports. Class A: Clear evidence of internal mechanisms (validation of gene manipulation or receptor/host gene necessity); Class B: Preliminary evidence of *in vivo* relevance (animal studies are effective, and the mechanism is derived from biomarkers or *in vitro* intervention inferences); Class C: Limited to *in vitro* observation results.

### Pro-inflammatory roles and mechanisms of BEVs

BEVs contribute significantly to antibiotic resistance and systemic inflammation, complicating therapeutic interventions. A recent study by Burt *et al*. [75] demonstrated that OMVs from *Klebsiella pneumoniae* can bind to polymyxins, effectively acting as decoys that shield bacteria from antibiotic action. This protective mechanism is mediated primarily by lipid A, a component of LPS, which is enriched in OMVs. Under antibiotic stress, bacteria increase vesiculation and alter OMV lipid composition, enhancing their capacity to sequester antibiotics and promote bacterial survival [75].

Periodontal pathogen-derived OMVs are enriched with various virulence factors, such as muropeptides, LPS, fimbriae, gingipains, outer membrane proteins and proteases [134, 135]. These OMVs act as representatives of parent bacteria, facilitating communication with other oral bacteria and host cells and adhering to tooth surfaces [136]. Other species in oral biofilms, such as *Tannerella forsythia*, also release OMVs associated with biofilm formation. OMVs can accelerate the progression of periodontitis by protecting other microorganisms from complement activity. For instance, Aggregatibacter-derived OMVs serve as decoys, relying on LPS to activate and deplete complement, thereby shielding serum-sensitive bacteria. Furthermore, Pg OMVs induce selective TNF deficiency, impairing the recognition of microbes by macrophages/monocytes. Beyond evading innate immune surveillance, OMVs can also escape attacks from adaptive immune cells. For example, OMVs derived from Aggregatibacter, Pg and *Treponema denticola* carry sRNAs that inhibit IL-13 and IL-5 release in Jurkat T cells. These findings suggest that periodontal pathogen-derived OMVs promote bacterial survival and aggregation through multiple mechanisms, exacerbating the pathological processes of periodontitis [137].

OMVs can activate the first line of defense in oral mucosal epithelium through various pathways to resist bacterial infection [138, 139]. Pg OMVs are internalized by epithelial and endothelial cells via lipid raft-mediated endocytosis, facilitating the invasion of other pathogens such as *T. forsythia* [140]. After invasion, OMVs inhibit the migration and proliferation of oral epithelial cells, leading to dysfunction in periodontal tissue cells. For example, Pg OMVs, upon uptake by human periodontal ligament cells, activate the inflammasome, resulting in apoptosis and pyroptosis [141]. After breaching the oral epithelial barrier, OMVs enter submucosal tissues and directly interact with the host’s innate or adaptive immune cells. OMVs released by Pg, *T. forsythia* and *T. denticola* activate PRRs on macrophages and monocytes, increasing the production of TNF-α, IL-1β and IL-8.

Similarly, Aggregatibacter OMVs trigger inflammatory responses in monocytes by activating the NOD1-dependent NF-κB signaling pathway. Additionally, *F. nucleatum* OMVs promote macrophage polarization toward a pro-inflammatory phenotype [116]. OMVs may further exacerbate the inflammatory environment by impairing neutrophil chemotaxis and phagocytosis, thereby enhancing their toxicity to gingival fibroblasts and periodontal tissues. The main pathway for *F. nucleatum* OMVs to enter hPDLSCs is caveolin-dependent endocytosis, which is significantly different from the endocytosis mode of other pathogenic bacteria OMVs [142]. Internalized OMVs significantly inhibit the migration ability of hPDLSCs, while activating intracellular ROS generation [116, 143]. ROS, as a key upstream activator of the NLRP3 inflammasome, further amplifies the inflammatory cascade reaction [144].

Bacterial OMVs aggravate alveolar bone loss and tissue destruction by delivering toxic substances to susceptible cells in periodontal tissues. For instance, Aggregatibacter OMVs transmit cytotoxic distending toxins, promoting damage to human gingival fibroblasts and leading to the destruction of the junctional epithelium. Recent studies indicate that BEVs from both oral commensal bacteria and periodontal pathogens can trigger osteoclastogenic activity via TLR2 activation [145]. Moreover, Aggregatibacter OMVs directly induce alveolar bone loss by delivering PG. Similarly, *F. nucleatum* OMVs accelerate periodontal bone loss in a mouse periodontitis model by increasing osteoclast numbers and the production of inflammatory cytokines such as IL-1β, IL-6 and TNF-α [18]. In summary, periodontal pathogen-derived OMVs contribute to the formation of an inflammatory microenvironment and bone destruction in periodontal tissues by activating or degrading bioactive substances in host cells, inhibiting cell proliferation, promoting cell death and inducing the release of inflammatory cytokines.

Inhalation of organic dust, a significant occupational hazard in concentrated animal feeding operations (CAFOs), is associated with respiratory inflammation and chronic lung diseases. Bioaerosols generated in these environments contain high concentrations of bacteria and their byproducts, including BEVs. Recent studies suggest that BEVs play a critical role in triggering pulmonary inflammation and tissue remodeling, contributing to the pathogenesis of organic dust-related respiratory disorders. Treatment of airway epithelial cells, THP-1 monocytes and macrophages with dust OMVs rapidly induces the expression of IL-8, IL-6, ICAM-1, pro-IL-1β and TNF-α. In airway epithelial cells, the induction of inflammatory mediators is associated with elevated mRNA levels and NF-κB activation [146]. Polymyxin B fails to inhibit the induction of inflammatory mediators by dust OMVs. Single and repeated exposure to dust OMVs increases levels of KC, IL-6 and TNF-α in mouse lungs, without significantly altering IL-17A levels. The increase in cytokines correlates with enhanced neutrophil infiltration in the lungs. Repeated exposure to dust OMVs results in increased mean linear intercepts and peribronchial collagen deposition in mouse lungs, indicating pulmonary remodeling. Post-treatment, mice exhibit bronchial cellular infiltration and thickened airway epithelium. Due to their nanoscale size, OMVs can reach and accumulate in bronchioles and alveolar regions, causing lung damage and contributing to the development of respiratory diseases. These findings provide new evidence for the presence of BEVs in organic dust and their role in pulmonary inflammation and injury [147].

In addition to BEVs, host-derived EVs also contribute to rheumatoid arthritis pathogenesis. Irreversible tissue damage in RA, such as cartilage erosion, is attributed to the secretion of matrix-degrading enzymes by synovial fibroblasts, some of which are associated with EVs. Synovial fibroblasts from RA patients secrete vesicles with polymerase activity. Compared to healthy individuals, RA patients exhibit higher levels of platelet-derived microvesicles in their EVs, and platelet-derived microvesicles (PMVs) counts correlate with disease severity. In one study, PMVs and leukocyte-derived microvesicles were detected in the synovial fluid of RA patients. Many studies have reported a decline in PMV counts in the circulation and inflamed joints of RA patients. PMVs carry pro-inflammatory cytokines, and synovial fluid microvesicles can induce coagulation, leading to fibrin deposition and joint inflammation. The abundance of procoagulant microvesicles in RA patients may contribute to cardiovascular comorbidities, as platelet-derived extracellular vesicles (PDEVs) exhibit procoagulant and pro-inflammatory properties, impair endothelial protective functions and induce angiogenesis. Microvesicles isolated from the synovial fluid of RA patients have profound biological effects, including promoting the synthesis of B-cell activating factor and increasing the production of IL-8, CCL5 (RANTES), CCL2 (MCP1), IL-6, ICAM-1 and VEGF by cultured synovial cells. However, the centrifugation methods used in these studies may result in microvesicle samples contaminated with protein aggregates, immunoglobulins and complement proteins. In studies focusing on the effects of leukocyte-derived microvesicles on synovial cells, the risk of protein aggregate contamination in EVs is reduced. Platelet-derived microvesicles can also stimulate synovial cells via IL-1β. Microvesicles from T cells and monocytes activate multiple signaling pathways in synovial fibroblasts, including NF-κB, AP-1, p38 MAPK and JNK. Upon activation, synovial fibroblasts upregulate cyclooxygenase-2 and prostaglandin E synthase, induce the release of matrix metalloproteinases (MMPs), and increase the production of pro-angiogenic chemokines. In summary, microvesicles derived from platelets and leukocytes in the synovial fluid of RA patients play a key role in inflammation and local joint destruction by activating synovial fibroblasts. Additionally, increased EV secretion in RA patients may elevate the risk of cardiovascular disease. From a biomarker perspective, synovial microvesicle profiles are characteristic of RA; compared to patients with osteoarthritis (OA) or juvenile idiopathic arthritis (JIA), RA patients exhibit significantly higher numbers of CD3^-^CD8^+^ T-cell-derived microvesicles [148, 149].

### The immunomodulatory function of BEVs

The immunomodulatory effect of BEV is much more than the activation of the TLR2/4-NF-κB/MAPK pathway. Different BEVs carry different molecules, and host cells sense them through a variety of pattern recognition receptors, with widely varying effects. For example, some BEVs activate NOD2 via peptidoglycan [150], some activate endosome TLR9 via DNA [151] and some are recognized by C-type lectin receptors via surface polysaccharides, which do not cause inflammation but promote immune tolerance [152]. The content itself is also very particular: the lipopolysaccharide of the probiotic BEV, because of its different structure, only mildly induces IL-10. However, LPS from pathogenic bacteria will continuously activate NF-κB and trigger a strong inflammatory response. The same is true for lipoproteins and nucleic acids, where subtle differences in structure determine whether they are ultimately pro- or anti-inflammatory. For example, the LPS of probiotic bacteria such as *B. thetaiotaomicron* features a penta-acylated, monophosphorylated lipid A structure that elicits a markedly attenuated inflammatory response compared to pathogenic variants. Subtle variations in fatty acid composition and phosphate placement within the lipid A moiety can produce 100- to 1000-fold differences in LPS potency in a TLR4-dependent manner [153]. Similarly, the number and length of acyl chains within lipid A directly correlate with the capacity of LPS to activate TNF production by macrophages [154]. It can be said that BEV acts like an informative package, and the specific morphology of its contents directly determines how the host cell interprets it.

The regulation also shows strong cell and tissue specificity. When the same BEV encounters intestinal epithelial cells, macrophages or DCs, the response can differ substantially among cell types, as demonstrated by single-cell RNA sequencing and host-microbe protein-protein interaction network analyses showing that BEV targets differ among dendritic cells, macrophages and monocytes in a cell-type-specific manner [155]. For intestinal epithelial cells, it mainly helps to strengthen the barrier; macrophages induce anti-inflammatory cytokines such as IL-10 and establish immune tolerance. Cell-specific PRR expression explains much of this difference. Tissue microenvironments add further regulation. Mucus retention, hypoxic clearance and chronic exposure tolerance all contribute. These factors make BEV functions highly flexible [155].

Under pathological conditions, this fine regulatory network is often disrupted. The anti-inflammatory response of immune cells to BEV is significantly attenuated in inflammatory bowel disease (IBD) patients, while the macrophage response to BEV even changed from anti-inflammatory to pro-inflammatory in people with the NOD2 gene mutation. In the hepatocellular carcinoma (HCC) microenvironment, tumor-associated macrophages fail to respond to certain signaling pathways of BEV and become immunosuppressive. In addition, BEV can leave long-term effects through epigenetic and metabolic reprogramming, such as maintaining high expression of anti-inflammatory factors in some cells for weeks afterwards [128]. For instance, exposure to *P. aeruginosa* OMVs has been demonstrated to induce genome-wide loss of DNA methylation at enhancer and DNase hypersensitive site regions in human lung macrophages, indicating that BEVs can directly remodel the host epigenetic landscape [156]. Furthermore, BEVs transport substantial amounts of tRNA-derived fragments (tRFs) that can form stable secondary structures in the host cytoplasm and post-transcriptionally regulate immune gene expression, providing a mechanistic basis for the sustained anti-inflammatory phenotype [157]. The pathogenic and probiotic BEVs also have diametrically opposite effects on mitochondrial function, with one activating the inflammasome and the other suppressing excessive inflammation through mitophagy [158]. Mechanistically, pathogenic BEVs can trigger mitochondrial dysfunction that leads to K^+^ efflux and subsequent assembly of the NLRP3/NLRC4 inflammasome complexes, which activate caspase-1 and caspase-11 to drive pro-inflammatory cytokine release and pyroptosis. In contrast, probiotic BEVs have been shown to enhance mitophagy, the selective autophagic degradation of damaged mitochondria, and negatively regulate NLRP3 inflammasome activation and thereby restrain excessive inflammation [159]. These interconnected mechanisms form a complex network. This explains how BEVs can both drive and resolve inflammation [155].

## Application of BEVs in inflammatory diseases

BEVs have emerged as critical mediators of intercellular communication between the microbiota and the host, offering new opportunities for diagnosis and treatment of inflammatory diseases. In recent years, significant advances have been made in understanding the molecular composition, biogenesis, immunomodulatory functions and therapeutic applications of BEVs [160, 161]. BEVs exhibit dual roles in inflammatory diseases, acting either as anti-inflammatory mediators or pro-inflammatory triggers, depending on their origin, cargo, modification methods, diameters and mechanisms of action [128]. Researchers have demonstrated that BEVs derived from commensal and probiotic bacteria can enhance intestinal barrier integrity, stimulate regulatory immune responses and reduce pro-inflammatory cytokine production. Conversely, BEVs from pathogenic bacteria may exacerbate inflammation by engaging PRRs and activating inflammasome pathways [162]. Their unique properties, such as biocompatibility, natural targeting ability and immunomodulatory effects, make them promising candidates for diverse applications in disease treatment. To clarify the distinct roles of BEV subtypes, their primary mechanisms and inflammatory outcomes are summarized in Table 2.

**Table 2:** Subtype-specific mechanisms of BEVs in inflammatory regulation.

BEV subtype	Typical origin	Key cargo examples	Primary host pathways	Dominant inflammatory phenotype	Example
OMVs	Pathogenic G^-^ bacteria	Lipopolysaccharide (LPS), gingipains, FadA	TLR4/2 → NF-κB, MAPK, Inflammasome	Pro-inflammatory	Periodontitis [106], RA [52]
OMVs	Commensal G^-^ bacteria	Polysaccharide A (PSA), Sphingolipids	TLR2→ IL-10, Treg differentiation	Anti-inflammatory, Tolerogenic	Colitis [101, 136]
CMVs	Probiotic G^+^ bacteria	Lipoteichoic Acid (LTA), Cytoplasmic proteins, RNA	Endocytosis→M2 polarization, IL-10	Anti-inflammatory, Pro-reparative	Wound healing [27], Diabetes [163]
OIMVs	Pathogenic G^-^ bacteria	Cytoplasmic DNA/periplasmic enzymes	cGAS-STING (potential)	Potentially Pro-inflammatory	Biofilm-associated infections [66]

The therapeutic potential of BEVs extends far beyond metabolic regulation, with OMVs emerging as particularly versatile nanocarriers that demonstrate breakthrough capabilities across multiple therapeutic domains. Engineered OMV systems exhibit remarkable dual functionality, positioning them as next-generation multimodal nanoplatforms capable of addressing two critical healthcare challenges: cancer therapy and antimicrobial resistance. In targeted oncology therapeutics (Figure 3A), DOX-loaded OMVs derived from attenuated *K. pneumoniae* demonstrate rapid cellular uptake in NSCLC A549 cells, potent tumoricidal activity (*in vitro* and *in vivo*) and innate immunostimulatory properties through tumor-associated macrophage (TAM) recruitment, enabling synergistic chemoimmunotherapeutic effects [164]. Simultaneously, in precision anti-infective delivery, as shown in Figure 3B, antibiotic-encapsulated OMVs achieve localized intestinal drug retention (>36 h) with minimal systemic exposure, significantly enhancing pathogen clearance while potentially circumventing antibiotic resistance development [168]. This dual therapeutic paradigm establishes OMVs as a transformative platform for next-generation nanomedicine, bridging critical gaps in both oncological and anti-infective therapies. Figure 3C showcases a pH-responsive (6.8) combinatorial nanotherapy system that orchestrates sequential release of paclitaxel (PTX) and Redd1-siRNA, demonstrating remarkable multifactorial efficacy in triple-negative breast cancer—achieving 68% tumor suppression through PTX-mediated M2 macrophage glycolytic activation and subsequent TAM repolarization, while simultaneously remodeling the immune microenvironment and inhibiting metastasis [166]. Moreover, parallel innovations in genetic engineering have produced scalable tRNA-pre-miRNA OMV platforms, as illustrated in Figure 3D. These platforms leverage a unique tRNA scaffold mechanism to achieve 82% CXCR4 oncogene silencing through efficient eukaryotic processing of pre-miR-126, delivering potent antiproliferative effects in breast cancer models [167]. These advances move beyond passive drug carriers. New systems offer stimulus-responsive release, genetic modulation and microenvironment engineering. OMVs are now versatile platforms for next-generation therapeutics.

**Figure 3:**
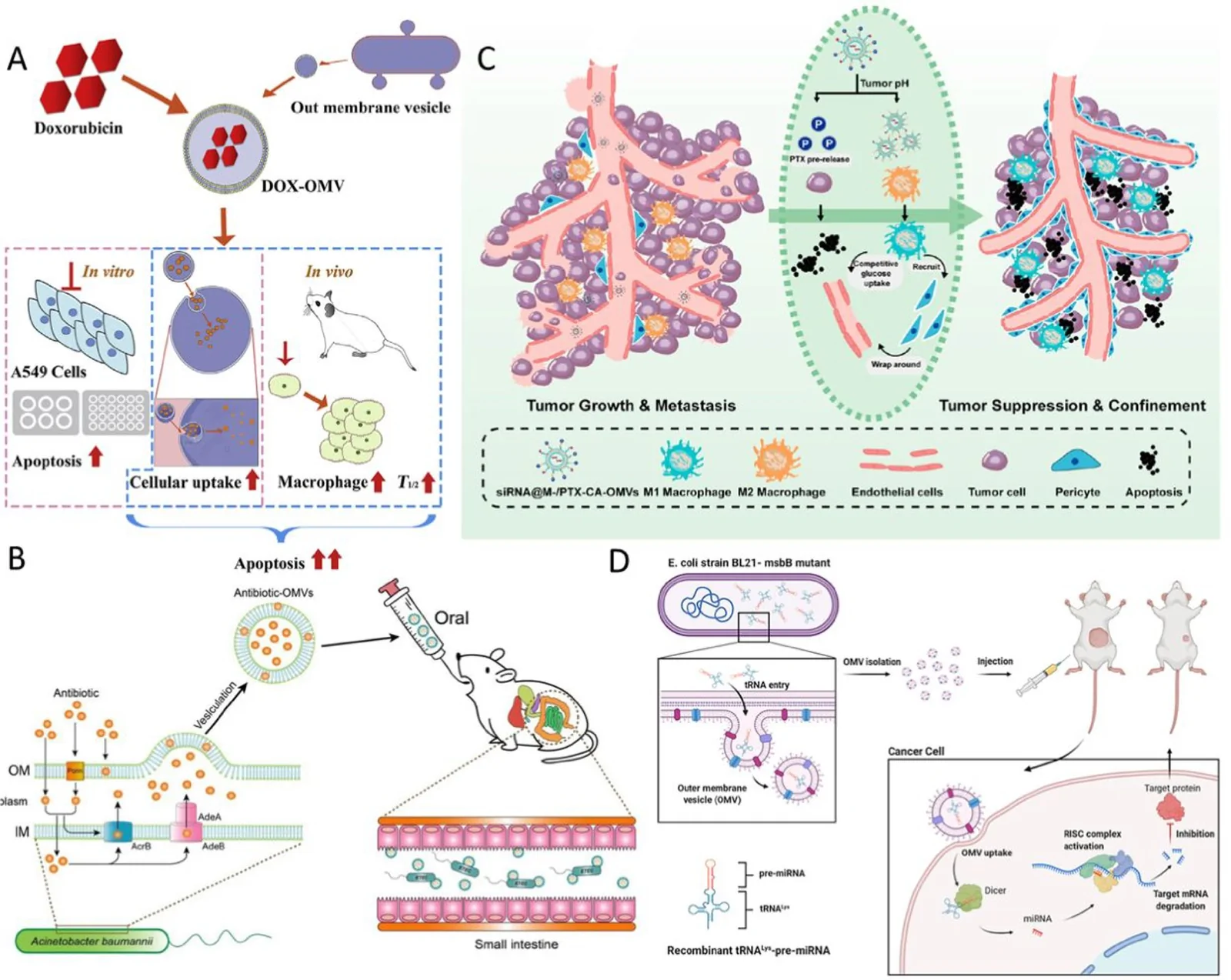
BEVs in cancer therapy and inflammation: (**A**) overview of anti-NSCLC effect triggered by DOX-OMV. Reprinted with permission [164]. Copyright 2020, The Author(s). (**B**) Schematic diagram of the design of novel nanoantibiotics using an OMV-based drug efflux mechanism. Reprinted with permission [165]. Copyright 2019 Elsevier B.V. All rights reserved. (**C**) The impact of BEVs in tumor growth, metastasis and confinement. Reprinted with permission [166]. Copyright 2021, The Author(s). (**D**) Schematic illustration of the bacterial OMV over-expressed pre-miRNA as a delivery vehicle for 4T1 breast cancer therapy. Reprinted with permission [167]. Copyright 2022, The Author(s).

### Anti-inflammatory application

IBD is one of the most extensively studied conditions with respect to BEV applications. BEVs isolated from gut commensal bacteria, particularly from *B. thetaiotaomicron* and *Bacteroides fragilis*, have demonstrated promising anti-inflammatory effects in several murine models of colitis. Administration of these BEVs has been associated with reductions in colonic inflammation, improved epithelial barrier function and modulation of cytokine profiles toward an anti-inflammatory state, marked by increased IL-10 secretion and suppression of TNF-α and IL-1β [155, 169–171]. In addition, BEVs have been shown to condition bone marrow-derived monocytes to produce a higher IL-10/TNF-α ratio upon LPS challenge, suggesting long-term modulation of innate immune responses that may protect against recurrent inflammatory insults [128].

Recent studies have further explored the use of BEVs as delivery vehicles for therapeutic agents in IBD. For example, BEVs derived from *E. coli* Nissle 1917 have been engineered to encapsulate anti-inflammatory agents and demonstrate improved targeting to inflamed colonic tissues, leading to attenuation of experimentally induced colitis [161]. The incorporation of nanotechnology has enabled the development of ‘nanoprobiotics’ in which BEVs are encapsulated with nanoparticles to enhance their stability, bioavailability and targeted delivery to inflamed regions of the gut [169]. Such approaches have shown increased efficacy in reducing oxidative stress and inflammatory cytokine production, suggesting that BEVs may provide a minimally invasive alternative to conventional IBD therapies [172]. For instance, a very recent study by Chen *et al*. [173] demonstrated that *Lactobacillus plantarum* EVs restore the intestinal barrier, drive macrophage polarization toward M2 and markedly increase IL‑10 and TGF‑β while reducing pro‑inflammatory cytokines in DSS colitis, with clear improvement in disease scores and histology.

In periodontitis, BEVs derived from periodontal pathogens and commensal species have been implicated in modulating local immune responses. While pathogenic BEVs from bacteria such as *P. gingivalis* are known to promote inflammatory cascades leading to tissue damage [174], beneficial BEVs from commensal species have the potential to counteract these effects by modulating dendritic cell responses and regulating cytokine production [172, 175]. These findings raise the possibility of using engineered BEVs as therapeutic agents for periodontal regeneration and in reducing the inflammatory burden associated with chronic periodontitis [175, 176]. In periodontitis treatment, BEVs from *P. gingivalis* (Pg-EVs) exhibit vaccine potential. Pg-EVs are structurally and functionally stable. They resist proteinase K degradation. They deliver bacterial components to the host immune system. Intranasal immunization with OMVs combined with the TLR3 agonist Poly(I: C) significantly reduced *P. gingivalis* colonization while elevating IgG (including IgG1 and IgG2a) and s-IgA levels in saliva and serum. Pg-EV safety was confirmed via intracerebral injection, with further studies indicating their strong immunogenicity and antigenicity, likely attributable to LPS and A-LPS-modified proteins in OMVs [177]. Thus, Pg-EVs or OMVs from other periodontal pathogens demonstrate potential as periodontitis therapeutic vaccines, offering bacterial clearance, safety, low cost, high efficacy and ease of transport. The ability to simultaneously deliver antigens and modulate immune responses positions BEVs as truly versatile platforms capable of addressing diverse medical challenges through a unified nanotechnology approach.

Beyond gastrointestinal and periodontal inflammation, BEVs have also been explored in the context of metabolic and systemic inflammatory diseases. BEVs from Bacteroides species have been shown to enhance gut barrier integrity and modulate systemic metabolic responses, thereby ameliorating conditions such as nonalcoholic fatty liver disease (NAFLD) and type 2 diabetes mellitus (T2DM) [176]. These vesicles, often rich in enzymes and bioactive lipids, contribute to improved lipid metabolism and reduced systemic inflammation, which are critical for maintaining metabolic homeostasis in chronic inflammatory states [176, 178].

Neuroinflammatory conditions, including Alzheimer’s disease (AD) and Parkinson’s disease (PD), represent another frontier for BEV application. BEVs are capable of crossing the blood–brain barrier (BBB) and modulating neuronal and glial functions. Research has demonstrated that BEVs derived from both pathogenic and probiotic sources can influence neuroinflammatory processes: while BEVs from pathogenic bacteria may activate microglia and astrocytes to produce pro-inflammatory mediators [179], BEVs from probiotic bacteria such as Lactobacillus have been associated with neuroprotective effects through the promotion of M2 macrophage phenotypes and increased production of brain-derived neurotrophic factor (BDNF) [179]. These dual roles illustrate the potential for employing BEVs in designing novel nanotherapeutic approaches aimed at reducing neuroinflammation and protecting neuronal integrity [179].

BEVs have also been leveraged for their diagnostic potential in inflammatory diseases. Recent pilot studies have demonstrated that BEVs circulating in blood or present in other body fluids can serve as biomarkers indicative of underlying inflammatory states. For instance, plasma-derived BEVs have been profiled in patients with IBD, revealing distinct differences in microbial diversity and EV cargo when compared to healthy individuals. Such findings suggest that BEV profiling may aid in the early diagnosis of inflammatory diseases and in monitoring treatment responses [129, 180].

Endometriosis, a chronic inflammatory condition affecting reproductive tissues, has also been linked to altered BEV profiles. Recent studies have shown that BEVs in endometriosis patients carry elevated levels of pro-inflammatory cytokines that may contribute to disease progression and pain. Moreover, alterations in the cargo composition of BEVs from both host and bacterial origins have been implicated in promoting aberrant tissue remodeling and angiogenesis within endometrial lesions, suggesting that BEVs could serve as potential biomarkers and therapeutic targets in endometriosis [181].

Another promising application area involves the use of BEVs to modulate the gut-brain axis. As key mediators of interkingdom communication, BEVs derived from gut microbiota influence neural signaling, stress responses and cognitive functions. Preclinical evidence indicates that BEVs can ameliorate depressive and anxiety-like behaviors in animal models by modulating neuroinflammatory pathways and influencing central cytokine production [179]. These findings open new avenues for leveraging BEVs as adjunctive therapies in neuropsychiatric disorders with an inflammatory component.

In summary, BEVs represent a novel and versatile platform for the modulation of inflammatory responses, offering opportunities not only to alleviate chronic inflammation but also to restore tissue homeostasis and protect against disease progression. Ongoing research continues to reveal new insights into their molecular mechanisms and therapeutic applications, paving the way for future clinical trials and eventual integration into the standard of care for inflammatory diseases [80].

### Pro-inflammatory applications

OMVs exhibit a dual role in inflammatory processes, serving as both mediators of pathological inflammation and platforms for innovative therapies. While native OMVs can induce severe inflammatory responses through the release of pro-inflammatory cytokines (IL-6, IL-8, IL-1β, TNF-α) that cause vascular dysfunction and tissue damage [6], recent advances in bioengineering have transformed these vesicles into powerful therapeutic tools.

Inflammation plays a critical role in tumor initiation and progression, making it a key therapeutic target. BEVs, owing to their strong pro-inflammatory properties, can activate host immunity and enhance immune cell infiltration into tumors, showing great potential in cancer immunotherapy. Ban *et al*. [182] developed a microbial nanocomplex by encapsulating oncolytic viruses (OVs) into calcium phosphate (CaP)-coated, pyranose oxidase (P_2_O)-engineered OMVs, thereby enhancing autophagy-mediated antitumor immunity (Figure 4A). The CaP shell protects OVs from immune clearance and improves tumor accumulation. Inside tumor cells, P2O generates ROS from glucose, promoting autophagy and viral replication, ultimately inducing cancer cell death. This strategy leverages OMV-mediated tumor microenvironment (TME) modulation to boost oncolytic efficacy. BEVs have also emerged as promising immunoadjuvants in tumor vaccine development. Zhang *et al*. [183] constructed HMVs by fusing cytomegalovirus-derived membranes expressing viral antigens with bacterial OMVs (Figure 4B). These HMVs, carrying native spike proteins and PAMPs, enhanced DC uptake and antigen presentation, markedly boosting B-cell activation, T-cell proliferation, DC maturation and antigen-specific antibody production in mice.

**Figure 4:**
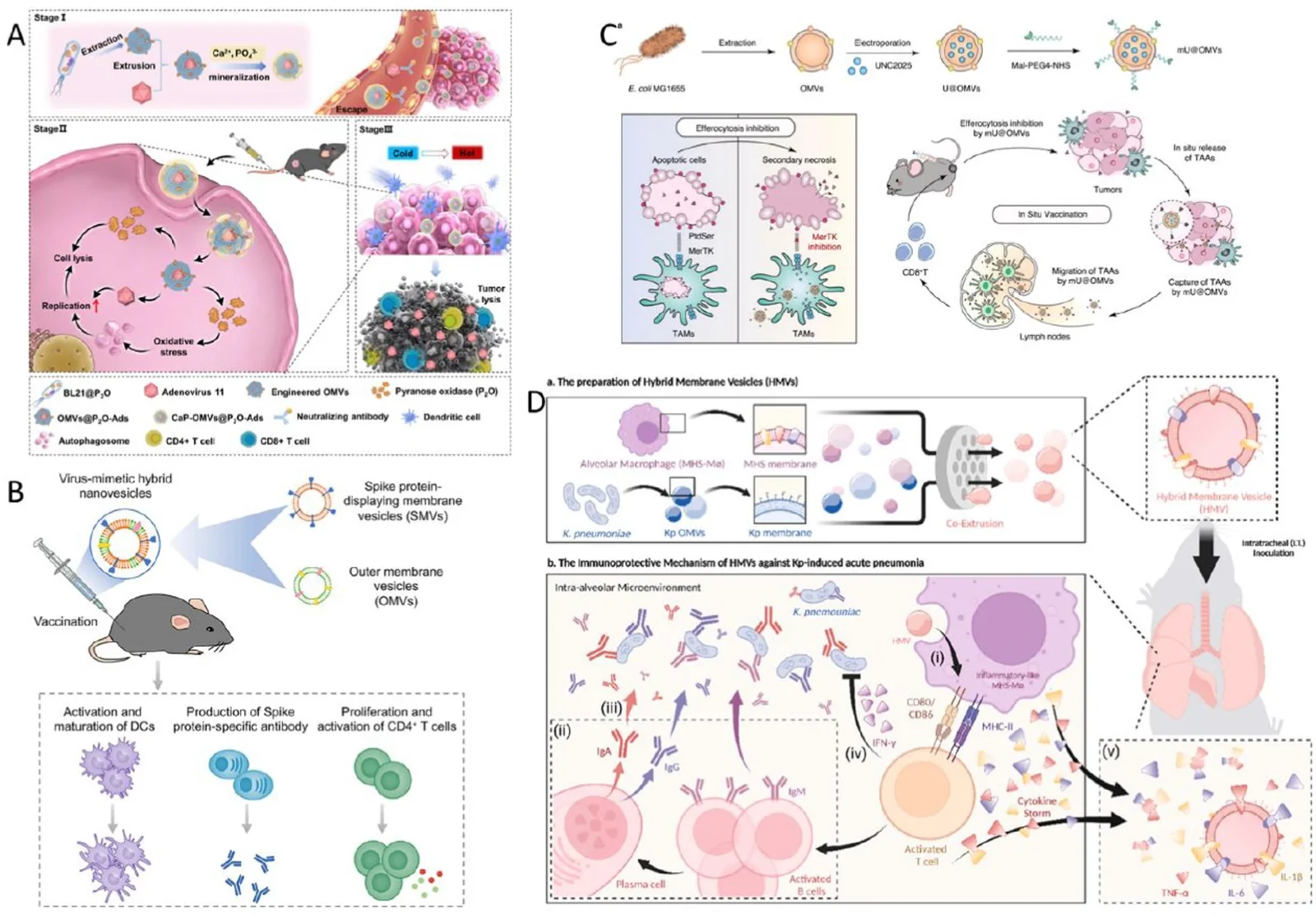
BEVs in tumor therapy and immunization: (**A**) the biomineralized microbial nanocomposite engineered from OVs for autophagy-cascade-augmented immunotherapy. Reprinted with permission [182]. Copyright 2023, Springer Nature BV. (**B**) The development of virus-mimetic hybrid nanovesicles (HMVs) for vaccination, where spike protein-displaying SMVs and OMVs activate DCs and induce antibody production and T cell proliferation. Reprinted with permission [183]. Copyright 2022, The Author(s). (**C**) Preparation and therapeutic strategy of mU@OMVs for *in situ* cancer vaccination. Reprinted with permission [184]. Copyright 2023, The Author(s). (**D**) The preparation and the immune-protective mechanism of HMVs. Reprinted with permission [185]. Copyright 2024, The Author(s).

Recent studies have combined BEVs with other therapeutic modalities to enhance antitumor responses. Zhuang *et al*. [184] developed a multifunctional OMV-based platform integrating MerTK inhibition and antigen delivery (Figure 4C). The MerTK inhibitor UNC2025 was encapsulated in maleimide-modified OMVs (mU@OMVs), which were engulfed by TAMs upon peritumoral injection. This blocked MerTK phosphorylation, induced secondary necrosis and enabled OMVs to capture tumor-associated antigens (TAAs) via thioether bonds. The co-delivery of TAAs and OMVs to lymph nodes stimulated potent CD8^+^ T cell responses, effectively suppressing tumor growth, metastasis and recurrence. This synergistic immune activation strategy has also been applied to infectious disease prevention. Jiang *et al*. [185] engineered HMVs by fusing *K. pneumoniae* OMVs with alveolar macrophage membranes for pulmonary immunization (Figure 4D). Administered via intratracheal instillation to mimic inhaled vaccines, HMVs rapidly recruited lung immune cells, induced pathogen-specific responses and established local mucosal immunity. In acute pneumonia mouse models (strains 43816 and 729088), HMV immunization significantly reduced bacterial load, improved survival and alleviated inflammation.

These OMV-based strategies demonstrate the vast potential of engineered BEVs in immunotherapy, vaccine development and targeted delivery. However, challenges remain. Gram-negative bacterial vesicles contain virulence factors and can trigger systemic inflammatory response syndrome (SIRS), characterized by leukopenia, thrombocytopenia and elevated IL-6 and TNF-α levels. Studies show that blocking IL-6 or TNF-α improves survival in septic patients. Using EV-based screening, salbutamol, a β2-adrenergic agonist and nortriptyline, a tricyclic antidepressant were identified as effective inhibitors of IL-6 and TNF-α release in EV-stimulated macrophages. In sublethal SIRS models, salbutamol and nortriptyline significantly reduced inflammatory cytokine levels in a dose-dependent manner, and their combination synergistically suppressed IL-6 and TNF-α secretion.

BEV inhibitors offer a promising strategy for treating bacterial infections and systemic inflammatory response syndrome (SIRS). Various chemical and natural compounds have shown inhibitory activity against BEV formation, release, host cell uptake and inflammatory responses [11]. These inhibitors not only function independently but can also be used alongside antibiotics to enhance efficacy and reduce resistance. Targeting BEVs is particularly crucial due to their multifaceted role in infection [10, 87, 186]. Gut microbes utilize BEVs to impair host defenses by neutralizing antimicrobial peptides (AMPs), promoting biofilm formation and facilitating toxin delivery [187]. For instance, *V. cholerae* OMVs transport cholera toxin (CT) into intestinal epithelial cells via GM1 receptor-mediated uptake, elevating intracellular cAMP [188]. Similarly, Listeria monocytogenes uses cytolysin-bearing CMVs to exert cytotoxic effects. Nonetheless, BEVs can also enhance host defense. OMVs deliver LPS to macrophages, activating caspase-11-dependent responses that promote bacterial clearance. Pretreatment with *E. coli* C25-derived OMVs has been shown to reduce bacterial internalization by inducing protective pro-inflammatory responses in epithelial cells.

BEVs exhibit dual roles in inflammatory diseases. Pathogenic BEVs exacerbate inflammation via TLR/NF-κB pathways, contributing to diseases such as periodontitis and rheumatoid arthritis. In contrast, BEVs from commensals or probiotics can attenuate inflammation by promoting M2 macrophage polarization, enhancing epithelial barrier function and maintaining microbiota balance. As natural nanocarriers, BEVs hold promise in vaccine development like meningococcal vaccines and drug delivery. However, clinical translation faces challenges, including heterogeneity, targeting efficiency, large-scale production and long-term safety. Future research should focus on optimizing BEV isolation and engineering, exploring synergistic functions of BEV subtypes and validating therapeutic efficacy in clinical trials to advance their application in precision medicine (Table 3).

**Table 3:** Application of BEVs in inflammatory diseases.

BEVs origin	Modifying method	Diameter	Effect	Mechanism	Disease	Ref.
*E. coli.*	Genetic engineering	220 nm	Promote inflammatory	Biomineral shells mask surface antigens of OMVs. Overexpressed Pyranose Oxidase (P_2_O) catalyzes the accumulation of excess H_2_O_2_ within tumor cells. High oxidative stress triggers cellular autophagy.	Cancer	[182]
*Salmonella*	Genetic engineering Endogenous modification	167.5 ± 2.3 nm	Promote inflammatory	Intact viral antigenic proteins with natural conformation bound to the lipid bilayer and Pathogen-Associated Molecular Patterns (PAMPs) promote antigen-presenting capability of DCs.	SARS-CoV-2	[183]
*E. coli.*	Genetic engineering Exogenous modification	100.04 ± 1.70 nm	Promote inflammatory	Prevent MerTK phosphorylation in tumor-associated macrophages (TAMs). Captures released antigens via the formation of universal thioether bonds. Utilize the intrinsic features of OMVs for lymph node targeting.	Cancer	[184]
*Klebsiella pneumoniae*	Genetic engineering	—	Promote inflammatory	Rapid mobilization of immune cells. Promotion of pathogen-specific immunity. Establishment of local mucosal immunity.	Acute pneumonia	[185]
*Klebsiella pneumonia*	Genetic engineering Exogenous modification	93.09 nm	Anti-inflammatory	Induces substantial tumor growth inhibition. Prime the immune system. Enhances the overall antitumor efficacy of Doxorubicin (DOX).	Non-small-cell lung cancer	[164]
*Acinetobacter baumannii*	Genetic engineering Exogenous modification	100–300 nm	Anti-inflammatory	Bacteria excrete vesicles via high efflux pump expression coupled with OMV secretion. Exerts passive and active bactericidal effects.	Intestinal infection	[165]
*E. coli.*	Genetic engineering Exogenous modification Endogenous modification	130 ± 15.6 nm	Anti-inflammatory	Dual regulation of the tumor microenvironment. Inhibition of tumor progression Suppression of tumor metastasis.	Cancer	[166]
*E. coli*	Genetic engineering Exogenous modification Endogenous modification	108.2 nm	Anti-inflammatory	Utilization of a ‘tRNA scaffold’ camouflage. Significant inhibition of CXCR4 expression in breast cancer tissues. Significant restraint of tumor proliferation.	Breast cancer	[167]
*S. aureus*	Exogenous modification	98.07 ± 0.21 nm, 95.77 ± 1.45 nm, 108.65 ± 0.96 nm	Promote inflammatory	Intracellular Delivery. Activation of proteasome-dependent antigen presentation pathways. Generation of subsequent robust CD8+ T cell responses.	Complications associated with *S. aureus* infection	[189]
*E. coli*	Genetic engineering Exogenous modification Endogenous modification	94.54 ± 1.46 nm	Anti-inflammatory	Induction of photothermal-photodynamic responses. Activation of TRAIL-induced apoptosis.	Melanoma	[190]
*Akkermansia muciniphila*	Genetic engineering Exogenous modification Endogenous modification	170.7 ± 1.9 nm	Promote inflammatory	Lipo@HEV penetrates into lymph node to initiate DC maturation and CTL activation, synergistically inhibiting the established tumor.	Cancer	[191]
*E. coli.*	Genetic engineering Exogenous modification Endogenous modification	170.6 nm	Anti-inflammatory	Activation of the BMP/SMAD signaling pathway. Enhances osteogenic differentiation	Osteoporosis	[168]
*Lactococcus*	Genetic engineering Exogenous modification Endogenous modification	—	Anti-inflammatory	VEGF-Mediated Endothelial Activation Lactate-Induced Immunomodulation Suppresses adipogenic differentiation	Diabetic	[192]
*E. coli.*	Genetic engineering	180 nm	Promote inflammatory	HPDA@ [OMV-CC] NP demonstrates uniform melanoma targeting capability and immune response activation by rapidly stimulating DC maturation in lymph nodes	Melanoma	[193]

## Clinical translation and challenges

The clinical translation of BEVs as therapeutic agents faces several significant challenges, primarily due to their heterogeneity, cargo loading and delivery, bioavailability, scalability and ethical considerations [194]. BEVs are highly diverse in size, cargo, membrane composition and biological function, which complicates their isolation, characterization and standardization [195]. This variability impedes reproducibility and accurate data interpretation, hindering the development of reliable therapies [195, 196]. Additionally, efficiently loading therapeutic cargos into BEVs and ensuring controlled release at target sites is challenging [197]. Innovative delivery methods such as 3D bioprinting show promise, but targeted delivery remains an issue as EVs can be taken up by various cell types [198].

Bioavailability presents another obstacle. BEVs are rapidly cleared by the immune system after administration. Enzymatic degradation further limits their stability and effectiveness [199]. Scalable production methods are also needed to meet clinical demands, as current culture systems are limited to two-dimensional static flasks [200]. Advances in 3D cell culture and dynamic bioreactor systems may address these scalability issues. Ethical concerns related to sourcing biological materials from donors and ensuring proper consent further complicate clinical applications, especially given the lack of a universally accepted international directive for EV use in clinical studies [201].

Many BEV activities are hybrid: the membrane can determine uptake route and endosomal escape, enabling cargo delivery, while cargo can tune the magnitude or direction of PRR-driven responses. In our opinion, when the mechanisms of loading effect and carrier effect are not yet fully understood, the key issue in drug design is not the cargo or the membrane, but rather, what is the dominant controllable knob under a certain therapeutic endpoint? Practical discriminators include biochemical protection assays to localize active principles to lumen vs surface [202]; host receptor perturbation to test membrane-PAMP dependence [160, 203]; and composition engineering to shift signaling without changing cargo identity [204]. Reviews caution that purification can leave contaminants and that genetic deletions may confound fitness or vesiculation, underscoring the need for orthogonal controls and potency assays tied to mechanism [202].

As a regenerative biomaterial, the clinical translation challenges of BEVs are more complex than those of common mammalian extracellular vesicles. Three core challenges are reflected: the endotoxin problem, the drift of immunogenicity caused by engineering, and the regulatory dilemma caused by cross-species sources. The LPS carried by BEVs is not only the functional basis of BEV but also the source of its toxicity. This homology of function and toxicity property poses a dilemma for drug development. Conventional methods poorly remove membrane-integrated LPS. Strong detergents destroy vesicle structure. Polymyxin affinity chromatography removes less than 20%. Ultrafiltration and size exclusion are largely ineffective. More troublesome is that the structure of LPS from different strains is different, and improper processing will either sacrifice the activity or cause instability from batch to batch. Therefore, it is necessary to redefine the standard of endotoxin in quality control and combine the limbocyte lysate method with the TLR4 reporter gene detection at the cellular level to establish the limit standard of ‘functional endotoxin unit’.

Functional readouts attributed to BEVs can be confounded by ‘pseudo-effects’ from endotoxin that is free in solution or co-purified with vesicles, other co-isolated soluble bacterial products or viable bacteria carried over into the vesicle preparation. Because OMVs intrinsically carry potent pathogen-associated molecular patterns, particularly LPS, many host responses can be driven by TLR4 signaling unless explicitly controlled [205, 206]. The controls below are widely recommended to distinguish vesicle-intrinsic activities from these artifacts (Table 4).

**Table 4:** Key control experiments to distinguish true vesicular functions from artifacts.

Contamination/pseudo-effect addressed	What the control measures	How to run/typical implementation	Interpretation if control is positive	Interpretation if control is negative	Key caveats/limitations
Limulus amebocyte lysate test	Enzymatic clotting or chromogenic cascade triggered by Factor C in the presence of endotoxins [207]	Use chromogenic or gel-clot LAL kits on vesicle preparations, comparing against a buffer blank; often involves dilution to fit a standard curve [208]	Endotoxin is present in the preparation, which may drive observed inflammatory effects rather than vesicle-specific cargo [208]	Preparation has low/negligible endotoxin levels; observed effects are likely independent of standard endotoxin	Susceptible to nanoparticle interference/masking; detects LPS presence but cannot distinguish biologically active from inactive LPS structures [207, 208]
TLR4 reporter assay	Functional biological activation of the TLR4/MD2/CD14 receptor complex leading to downstream signaling [207]	Expose engineered reporter cells to EV preparations and measure a reporter signal; use purified LPS as a positive control [145, 207]	The preparation contains biologically active TLR4 agonists capable of inducing an immune response [207]	The preparation lacks functional TLR4 agonists; immune responses observed in complex primary cells likely stem from other PAMPs/vesicle components [207]	Relies on engineered cell lines which may lack the full physiological complexity or alternative receptors of primary immune cells; free LPS vs. OMV-bound LPS can behave differently [205, 207]
Polymyxin B treatment	Reduction or abrogation of biological effects following the binding and neutralization of LPS by the cationic peptide polymyxin B [205, 207]	Pretreat EV preparations or co-incubate cells with polymyxin B and compare functional readouts to untreated EVs [205]	The observed biological response is highly dependent on LPS/endotoxin engaging TLR4 [205]	The biological response is driven by vesicle components other than LPS [205]	Polymyxin B binding efficiency can be reduced by specific LPS structural modifications; it may not neutralize all diverse LPS chemotypes completely [207]
Sterility testing	Presence of living bacterial cells surviving the vesicle isolation process [208, 209]	Plate a fraction of the OMV filtrate/pellet on appropriate agar or inoculate into liquid growth medium and incubate for extended periods [208, 209]	Preparation is contaminated with live bacteria; functional effects cannot be reliably attributed solely to nonliving vesicles [208, 209]	Preparation is sterile; effects are due to the vesicles or co-isolated nonliving components [208, 209]	Does not rule out nonliving contaminants; requires utilizing the optimal growth medium/conditions for the specific parent bacterial strain [208]

Immunogenicity drift caused by engineering modification is another potential hazard. Whether it is drug delivery, surface modification or construction of eukaryotic/prokaryotic hybrid vesicles, it may change the original immune recognition characteristics of BEV or even induce unexpected immune responses. In terms of raw materials, cultivation conditions and plasmid stability of engineered strains can affect the yield and purity of BEV. At the product level, a multiparameter quality control system should be established for particle size, density, charge and drug loading efficiency. The stability assessment should take into account the mechanical stress caused by storage, *in vivo* environment and even atomization inhalation. Nonclinical safety evaluation also faces the issue of species selection, and humanized TLR4 mouse models or nonhuman primates may need to be considered. A pragmatic preclinical safety pipeline for BEV therapeutics could thus proceed in three sequential stages. First, GLP-compliant repeated-dose toxicology studies should be combined with multiplex cytokine profiling to map the systemic inflammatory landscape, as has been recommended for EV-based therapeutics in recent preclinical evaluation frameworks [210]. Second, given the central role of LPS-TLR4 interactions in BEV-mediated immunotoxicity, confirmatory immunotoxicity testing in humanized TLR4/MD-2 mice—which express human TLR4 and MD-2 from BAC transgenes on a mouse TLR4/MD-2 double-knockout background and enable species-specific TLR4 response discrimination—should be performed on the most promising candidates [211]. Third, for BEV formulations intended for systemic or mucosal administration, radiolabeled biodistribution studies are essential; recent work demonstrated that *E. coli* OMVs labeled with ^64^Cu can be quantitatively tracked in mice using PET/MRI imaging, with the highest organ uptake observed in the liver and spleen at 3, 6 and 12 h post-administration [212]. The breakthrough lies in multidimensional collaboration: establishing a new endotoxin release standard with function as the end point, developing a prediction platform for the immunogenicity of engineered BEV, promoting the dialogue between regulators and the academic community to form chemistry, manufacturing, and controls (CMC) guidelines for BEV products, and exploring a feasible path for registration of combined products. These problems require systematic solutions. BEV-based therapies can then transition from laboratory studies to clinical practice [213] (Table 5).

**Table 5:** Bacterial EV subtypes, cargo and expected host immune responses.

Vesicle subtype	Typical enriched cargo features	Dominant PRRs/pathways	Expected immune outputs
LPS-enriched classical OMVs	Surface-exposed LPS; outer-membrane proteins; periplasmic components	TLR4 activation with MyD88/TIRAP → NF-κB/MAPK and TRAM/TRIF → IRF3/type I IFN; potency consistent with LPS contribution to lethality and CD14 dependence	Strong pro-inflammatory cytokines and potentially type I IFN programs [160]
‘Typical’ budding OMVs that deliver peptidoglycan	PG fragments packaged/transported into host cells; outer membrane scaffold that enables entry	NOD1/NOD2 → RIP2 → NF-κB after OMV-mediated PG delivery; responses can be TLR-independent *in vivo* in some settings	NF-κB-driven epithelial inflammatory programs; innate and adaptive immune activation dependent on NOD1 [214, 215]
OMVs enriched for flagellin	Flagellin in/with OMVs	TLR5 → NF-κB/MAPK; NLRC4/caspase-1 activation when flagellin reaches cytosol	Pro-inflammatory cytokines; pyroptosis signatures when cytosolic sensing occurs [160]
OIMVs/double-membrane vesicles	Double bilayer; cytoplasmic material including DNA and ATP; inner-membrane and cytosolic proteins	Increased likelihood of endosomal TLR9 and cytosolic DNA sensors; also delivers other MAMPs that can co-stimulate TLR2/TLR4	More mixed innate profile: TLR activation plus nucleic-acid–linked inflammatory programs [160, 216]
Explosive lysis-derived vesicles	Highly heterogeneous; envelope fragments reassemble; can enclose released DNA and cytoplasmic material; higher nucleic-acid-positive fraction during lysis	Enriched nucleic acid cargo shifts sensing toward TLR9, TLR8, AIM2 inflammasome; cytosolic access can also enable caspase-4/5/11 → pyroptosis and downstream NLRP3; DNA can also drive cGAS-STING-IRF3 → IFN-β	Increased propensity for type I IFN and inflammasome-linked outcomes, relative to strictly budding OMVs; response breadth expected to be larger due to cytoplasmic cargo [160, 217, 218]
Gram-positive membrane vesicles	DNA, RNA, PG; lipoproteins	TLR2, TLR7/8/9, NOD2; MV cargo can also be consistent with NLRP3 engagement via lipoprotein-related pathways	Cytokine/chemokine release from epithelial cells; autophagy engagement during intracellular processing [219]
Engineered OMVs	Designed antigen display; retain intrinsic PAMPs unless detoxified	Can induce DC maturation and MyD88-dependent programming for cross-presentation; also inherently engage PRRs such as TLR2/4/5 and intracellular NOD/TLR9 depending on cargo	Self-adjuvanted innate activation that supports adaptive immunity [220]

To enhance the reproducibility and scientific rigor of BEV research, a minimum set of quality control standards should be applied across isolation, characterization, functional studies and data reporting. During sample preparation and purification, strain cultivation conditions, including origin, medium type, culture time distinguishing between log phase and stationary phase, pH and temperature, must be recorded to avoid vesicle release artifacts induced by nonphysiological stress. For vesicle separation, differential centrifugation, such as 10 000×*g* filtration followed by 100 000×*g* ultracentrifugation for at least 2 h or size exclusion chromatography, is recommended, while reliance on filtration alone should be avoided. Contamination controls are mandatory: a negative control consisting of sterile culture medium processed in parallel must be included, and bacterial lysis contamination should be verified by qPCR targeting genomic DNA or by Western blot for intracellular proteins such as GroEL or EF-Tu [221].

Basic characterization of BEVs requires three essential items. Morphology should be confirmed by transmission electron microscopy to reveal typical cup-shaped or spherical vesicles with a lipid bilayer. Particle size distribution, determined by nanoparticle tracking analysis or dynamic light scattering, should show a main peak between 20 and 200 nm, with a polydispersity index below 0.3 considered optimal [221]. Marker detection must include at least two BEV‑enriched components, such as OmpA, LPS or small RNAs, as positive markers, while the absence of intracellular protein contamination such as GroEL or ribosomal proteins should be verified as negative markers [201].

For sample analysis and credibility management, component identification by proteomics requires filtering out common contaminants like keratin and bovine serum albumin, and nucleic acid analysis should remove ribosomal RNA while verifying the specific packaging of small RNAs [222]. Reliability of findings should be labeled accordingly: high reliability for results verified by multiple independent experiments, specifically at least three, with no detection in negative controls; medium confidence for two-step verification where function is supported but with some fluctuations; and low confidence or suspected contamination for detections that occur only once or are also present in controls, which require annotation and cautious interpretation. Finally, data reporting and transparency demand full disclosure of methodological details, including centrifugation parameters, filter membrane pore size, storage conditions and number of freeze-thaw cycles, and raw data from sources such as mass spectrometry, nanoparticle tracking analysis or RNA-seq should be deposited in public databases such as Mass Spectrometry (ProteomeXchange) or the Sequence Read Archive.

## Summary and prospects

BEVs are nanoscale membrane-bound structures released by both Gram‑negative and Gram‑positive bacteria through outer membrane budding or cell lysis and include subtypes such as OMVs, CMVs and OIMVs [102, 220]. These vesicles transport a range of biomolecules like proteins, lipids and nucleic acids. They are vital for bacterial survival, immune modulation and material exchange [223]. BEVs play dual roles in inflammation. Pathogenic BEVs carry LPS and other virulence factors. They activate inflammatory pathways and induce cytokine release. This exacerbates tissue damage in periodontitis and rheumatoid arthritis [220]; conversely, BEVs from symbiotic or probiotic bacteria exert anti-inflammatory effects by promoting M2 macrophage polarization, enhancing tight junction protein expression and supporting microbiota balance [224]. For instance, BEVs from *Akkermansia muciniphila* alleviate colitis, while probiotic‑derived BEVs inhibit bone resorption [128].

The nanocarrier properties of BEVs make them highly promising for applications in vaccine development, targeted drug delivery and the treatment of inflammatory diseases [225]. OMV-based vaccines, such as the meningococcal vaccine, demonstrate strong immunogenic potential. Additionally, engineered BEVs show high efficacy and low toxicity in cancer and infection treatments [226]. Despite these advantages, several challenges hinder their clinical translation, including product heterogeneity, the need for optimized targeting specificity and biocompatibility, large-scale production and long-term safety data.

Three actionable research priorities emerge from the current state of the field. (i) Integrated manufacturing and Quality-by-Design (QbD) frameworks: To overcome BEV heterogeneity and enable scalable clinical production, closed-loop dynamic bioreactor systems must be paired with inline microfluidic separation and real-time process analytical technologies (PAT) to shift production from static flask-based cultures to GMP-compliant pipelines. The QbD approach, entailing systematic identification of critical quality attributes (CQAs), critical process parameters (CPPs) and in-process analytical methods, has been successfully applied to the purification of bacterial outer membrane vesicle-based vaccines (GMMA platform) and provides a regulatory-endorsed framework for BEV manufacturing development [227]. (ii) Target-specific delivery and conditional activation: Next-generation BEV engineering should move beyond bulk cargo loading toward conditional activation strategies such as protease-activatable surface ligands or pH-responsive linkers that confine therapeutic action to disease sites, thereby enhancing bioavailability and precisely controlling immunogenicity. (iii) Clinical validation focused on unique BEV properties: Clinical trials should prioritize therapeutic endpoints where BEVs offer functional advantages over synthetic nanocarriers, such as mucosal vaccination for respiratory pathogens or the restoration of homeostatic immune tone in chronic inflammatory disorders, validating both safety and unique efficacy in well-defined patient cohorts. As these three axes converge, BEV-based therapeutics may transition from proof-of-concept studies into a distinct class of biologics, where the informational complexity of the bacterial vesicle is no longer a manufacturing obstacle but a therapeutic asset.
